# MORC1 represses transposable elements in the mouse male germline

**DOI:** 10.1038/ncomms6795

**Published:** 2014-12-12

**Authors:** William A. Pastor, Hume Stroud, Kevin Nee, Wanlu Liu, Dubravka Pezic, Sergei Manakov, Serena A. Lee, Guillaume Moissiard, Natasha Zamudio, Déborah Bourc’his, Alexei A. Aravin, Amander T. Clark, Steven E. Jacobsen

**Affiliations:** 1 Department of Molecular, Cell and Developmental Biology, University of California Los Angeles, 4028 Terasaki Life Sciences Building, 610 Charles E. Young Drive East, Los Angeles, California 90095, USA; 2 Division of Biology and Biochemical Engineering, California Institute of Technology, 1200 E California Boulevard, Pasadena, California 91125, USA; 3 Unité de génétique et biologie du développement, Instititute Curie, CNRS UMR3215, INSERM U934, Paris 75005, France; 4 Eli and Edythe Broad Center of Regenerative Medicine and Stem Cell Research, University of California Los Angeles, 615 Charles E. Young Drive South, Los Angeles, California 90095, USA; 5 Howard Hughes Medical Institute, University of California Los Angeles, 675 Charles E. Young Drive South, Los Angeles, California 90095, USA

## Abstract

The Microrchidia (*Morc*) family of GHKL ATPases are present in a wide variety of prokaryotic and eukaryotic organisms but are of largely unknown function. Genetic screens in *Arabidopsis thaliana* have identified *Morc* genes as important repressors of transposons and other DNA-methylated and silent genes. MORC1-deficient mice were previously found to display male-specific germ cell loss and infertility. Here we show that MORC1 is responsible for transposon repression in the male germline in a pattern that is similar to that observed for germ cells deficient for the DNA methyltransferase homologue DNMT3L. *Morc1* mutants show highly localized defects in the establishment of DNA methylation at specific classes of transposons, and this is associated with failed transposon silencing at these sites. Our results identify MORC1 as an important new regulator of the epigenetic landscape of male germ cells during the period of global *de novo* methylation.

## Abstract (web summary)

The Microrchidia (Morc) family of GHKL ATPases are important repressors of transposons and other DNA-methylated and silent genes in *A. thaliana*. Here, the authors show that MORC1 is responsible for repression and methylation of specific classes of transposons in the mouse male germline.

## Graphical abstract

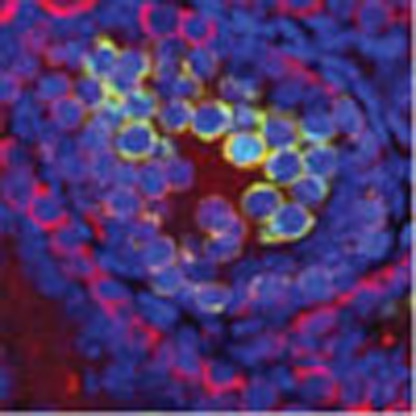

Two *Morc* genes in *A. thaliana*, *AtMorc1* and *AtMorc6*, were identified in forward genetic screens for novel transcriptional repressors^1^,^2^. AtMORC1 and AtMORC6 are required for silencing of a variety of transposons and are essential for higher-order chromatin compaction. The single *Morc* gene in *Caenorhabditis elegans* was also shown to be required for silencing of a repetitive transgene locus^1^. The founding member of the *Morc* gene family is mammalian *Morc1*. MORC1 is highly expressed in the blastocyst and male germline but is not expressed in most differentiated cells^3^. Mice deficient for MORC1 are normal, except that homozygous mutant males are infertile with small testicles (hence the name microrchidia)^4^,^5^. Male germ cells in the *Morc1* mutant do not undergo successful chromosomal pairing during the zygotene stage of meiosis and instead undergo apoptosis, with no germ cells surviving to complete prophase I.

During germ cell development, most DNA methylation is lost between E8.5 and E13.5. Then, between E13.5 and birth (∼E19), the genome undergoes global *de novo* methylation^6^,^7^,^8^. Failure to establish DNA methylation at this time causes transposon upregulation and meiotic failure. Indeed, the meiotic block in the *Morc1* mutant is similar to that observed for mice that have defects in DNA methylation and transposon repression, including mice deficient for DNA methyltransferases^9^,^10^,^11^ or the pre-meiotic Piwi-interacting RNA (piRNA) pathway^12^,^13^. Therefore, we hypothesized that MORC1 might be a critical factor for transposon silencing and DNA methylation in the mouse germline. Here we demonstrate that MORC1-deficient male germ cells undergo transposon derepression starting in late embryogenesis and continuing through the onset of meiosis. We also demonstrate that this phenotype is associated with failed locus-specific *de novo* methylation targeted specifically towards late-methylating transposon sequences.

## Results

### MORC1 represses transposons in the male germline

**Figure 1:**
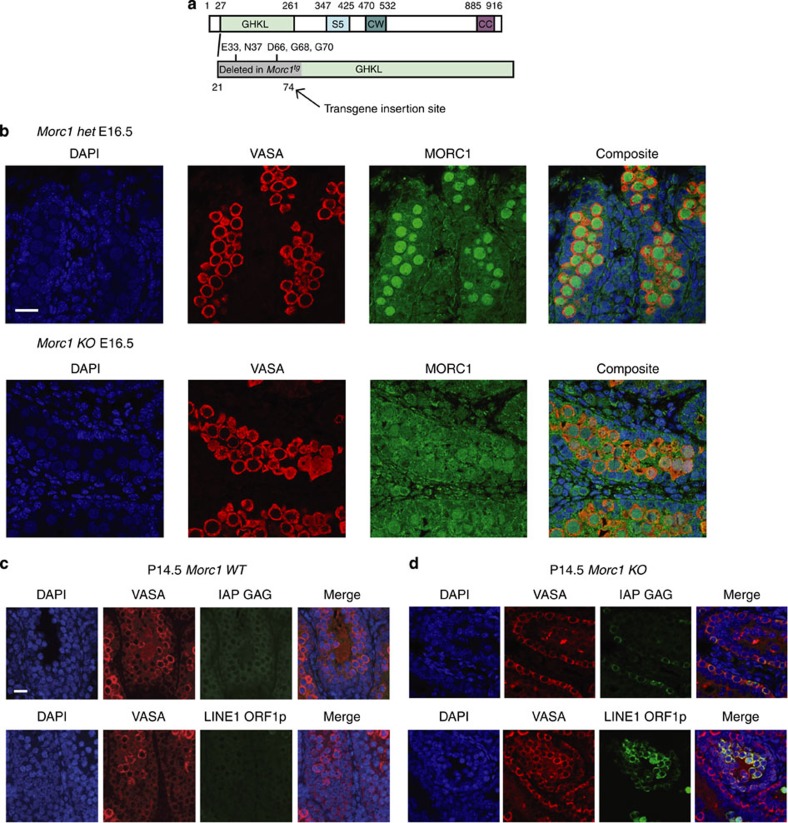
MORC1 is a nuclear protein essential for transposon repression. (**a**) Domain structure of *Morc1* gene and disruption in *Morc1*^*tg*^ allele. Deleted residues predicted to be critical for catalytic activity or ATP binding are denoted. (**b**) Detection of MORC1 by immunofluorescence (IF) in E16.5 testes. MORC1 is present as a germ cell-specific nuclear protein in the *Morc1*^*tg/+*^ (het) control but is absent from the *Morc1*^*tg/tg*^ (knockout (KO)). Aberrant expression of IAP GAG (**c**) and LINE ORF1p (**d**) in *Morc1*^*tg/tg*^ as detected by IF at P14.5. Note that IAP is overexpressed in most germ cells, whereas LINE is primarily present in the more differentiated cells deeper into the tubule. Scale bars, 20 μm (**b**–**d**).

To further characterize MORC1 we used a previously described FVB/N *Morc1* mutant (*Morc1*^*tg*^) mouse strain in which a tyrosinase gene was integrated into the *Morc1* locus^5^. Transgene insertion resulted in loss of exons 2–4, eliminating a large region of the GHKL ATPase domain including residues predicted to be critical for catalysis and ATP binding^14^ (Fig. 1a). Consistent with previous reports, we found that *Morc1*^*tg/tg*^ mice have a spermatogenesis defect with a complete absence of post-meiotic spermatids and spermatozoa (Supplementary Fig. 1a–c).

Quantative reverse transcription-PCR (qRT-PCR) from wild type (WT), embryonic whole testis indicates that *Morc1* messenger RNA becomes detectable at E14.5 and peaks at E16.5 (Supplementary Fig. 2a), which intriguingly is a period of rapid transposon methylation in the male germline. We generated an antibody against the coiled-coil domain of mouse MORC1 (Supplementary Fig. 2b) and found that MORC1 was localized to the nucleus of male germ cells at E16.5 (Fig. 1b). Conversely, MORC1 protein was undetectable in *Morc1*^*tg/tg*^ mutant germ cells (Fig. 1b). To test for transposon derepression in *Morc1*^*tg/tg*^ germ cells, we performed immunostaining for LINE1 ORF1p and intracisternal particle A (IAP) at postnatal day 14.5 (P14.5). We found that both of these transposon classes were ectopically expressed during early postnatal development, with particular enrichment of LINE1 ORF1p in the meiotic cells towards the centre of the tubule (Fig. 1c,d). LINE1 ORF1p was also derepressed at E16.5 and E18.5, showing that transposon derepression in *Morc1*^*tg/tg*^ arises well before the apparent meiotic defect (Supplementary Fig. 2c).

**Figure 2:**
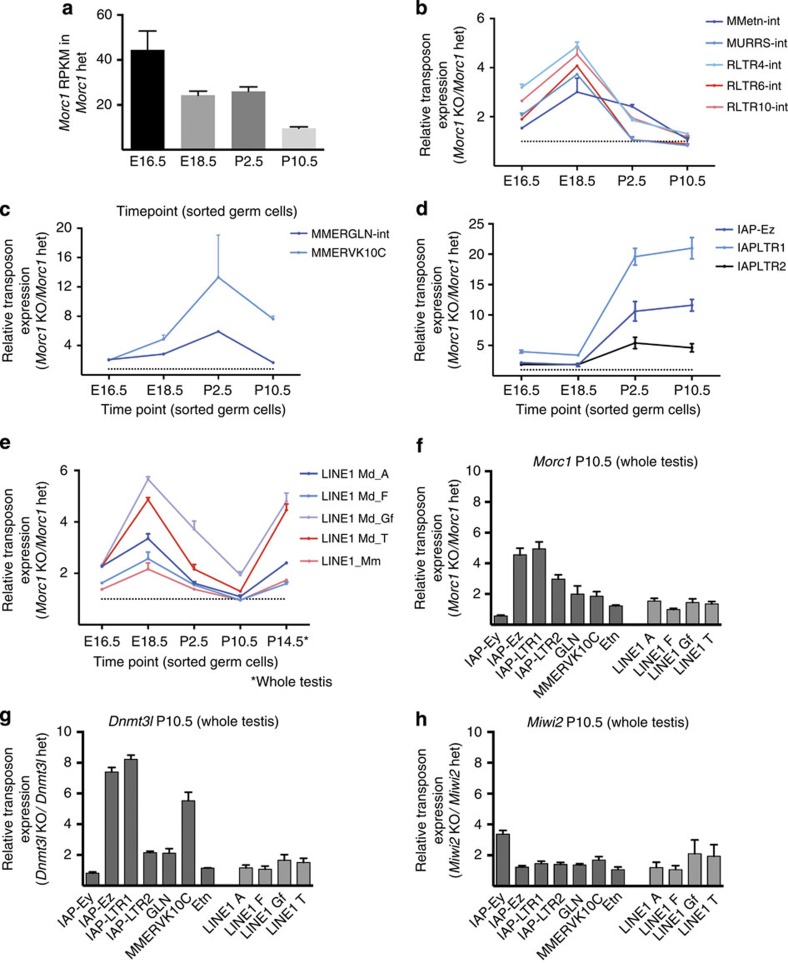
*Morc1*^*tg/tg*^ shows transposon upregulation resembling *Dnmt3l*^*−/−*^. (**a**) Expression of *Morc1* mRNA over development in *Morc1*^*tg/+*^, as measured by RNA-seq. (**b**–**e**) Overexpression of transposon species over the course of mammalian development, represented as a ratio of expression in the *Morc1*^*tg/tg*^ and the *Morc1*^*tg/+*^ control. Some LTR transposons show upregulation selectively in late embryogenesis (**b**), while others are overexpressed postnatally (**c**,**d**), and LINE elements are overexpressed both during late embryogenesis and again at the onset of meiosis (**e**). Overexpression of transposons in MORC1- (**f**), DNMT3L- (**g**) and MIWI2- (**h**) deficient whole testis. For **a**–**e**, the dotted line indicates a fold change of one. For **a**–**h**. Two to four replicates per genotype were analysed; all data are RNA-seq from sorted germ cells or whole testis as indicated. Mean+s.e. plotted.

To identify which genes and specific transposons are targets of MORC1-mediated repression, we performed mRNA-Sequencing (RNA-Seq) of whole testes from *Morc1*^*tg/tg*^ and *Morc1*^*tg/+*^ heterozygous controls at P10.5 and P14.5. Given that germ cells make up only a small percentage of the testis during the embryonic early and postnatal period, we also performed RNA-Seq on ribosomal RNA-depleted total RNA from sorted germ cells at E16.5, E18.5, P2.5 and P10.5. To purify germ cells, we crossed the *Morc1*^*tg*^ allele into the *Oct4*-*IRES-eGfp* reporter strain, which exhibits a distinct eGFP^+^ population from e9.5 to P2.5 (Supplementary Fig. 3a)^15^,^16^. To isolate germ cells at P10.5, we gated the side scatter (SSC) low (SSC^Lo^), epithelial cell adhesion molecule (EPCAM) high (EPCAM^Hi^) and major histocompatibility complex negative (MHCI^−^) population of testicular cells (Supplementary Fig. 3b). A list of sequencing samples and the experiments to which they correspond is included in Supplementary Data 1. Consistent with RT–PCR and published data^5^,^17^, *Morc1* mRNA was confirmed to show high expression at E16.5 and lower expression at later time points in the sorted germ cells (Fig. 2a).

The RNA-Seq data showed a broad transposon derepression defect in *Morc1*^*tg/tg*^ starting at E16.5 (Fig. 2c–e and Supplementary Data 2). Quantitative RT–PCR analysis of various transposable element classes gave similar results as RNA-Seq data (Supplementary Fig. 4a). In addition, RNA-Seq on sorted *Morc1*^*tg/tg*^ and control germ cells at P10.5 resembled the pattern of transposon derepression observed in whole testes (Supplementary Fig. 4a). Heterozygous *Morc1*^*tg/+*^ mice showed no marked increase in transposon expression relative to WT *Morc1*^*+/+*^ mice, confirming their validity as littermate controls (Supplementary Fig. 4b).

Different transposon classes showed different patterns of derepression in *Morc1*^*tg/tg*^. Some classes (RLTR4, RLTR6, MuRRS and Etn) were upregulated during embryogenesis but silenced even in the knockout at later time points (Fig. 2b). Other transposons (MMERVK10C, GLN and some IAP species) were most highly upregulated at postnatal time points (Fig. 2c,d). LINEs were upregulated both in late embryogenesis and again at P14.5 after the onset of meiosis (Fig. 2e), which was confirmed by immunofluorescence (Fig. 1d and Supplementary Fig. 2c). These fluctuations in transposon upregulation may reflect differences in the inherent transcriptional programmes of certain transposon classes, as well as varied effectiveness of other, partially redundant transposon repression pathways at different times. In the aggregate, however, these results indicate that MORC1 constitutes a new participant in transposon repression in the mammalian germline, acting on many different elements. Notably, MORC1 silences many transposon classes well after it is downregulated, consistent with it acting through an epigenetic mark such as DNA methylation.

### piRNA biogenesis occurs normally in the Morc1 mutant

During this period in germline development (E14.5 to birth), germ cells undergo mitotic arrest and global nuclear reprogramming that most notably involves genome-wide *de novo* DNA methylation mediated by the DNMT3A/DNMT3L complex^11^,^18^. The pre-meiotic piRNA pathway, involving the nuclear PIWI protein MIWI2, is also active during this period in promoting transposon silencing. To evaluate whether MORC1 acts on the same transposon classes as DNMT3L or MIWI2, we performed RNA-Seq on whole testes from *Dnmt3l^−/−^* (ref. 10) and *Miwi2^−/−^* (ref. 12) mice and their respective controls at P10.5, and compared this with the *Morc1*^*tg/tg*^ P10.5 whole testis data set. *Morc1*^*tg/tg*^ and *Dnmt3l*^*−/−*^ exhibited derepression of an overlapping set of transposons, primarily long terminal repeat (LTR) retrotransposons, while the *Miwi2*^*−/−*^ mutant testes had a milder phenotype and showed derepression of specific LINE elements and the IAP-Ey class of retrotransposons, which was not affected in *Morc1*^*tg/tg*^ or *Dnmt3l*^*−/−*^ mutant testes (Fig. 2f–h).

The lack of overlap between *Morc1* and *Miwi2* predicts that piRNAs would be unperturbed in *Morc1*^*tg/tg*^ mice. To test this, we performed small RNA sequencing of the testis at E16.5 to examine piRNA production. Our data revealed that the ratio of piRNA/microRNA and the generation of antisense piRNAs were unaltered in *Morc1*^*tg/tg*^ (Table 1), indicating that the piRNA pathway remains largely intact in *Morc1*^*tg/tg*^ testis at E16.5, and that transposon derepression in *Morc1*^*tg/tg*^ is most likely to be independent of the piRNA pathway. In fact, at P10.5 we observed an increase in the fraction of piRNAs derived from LTR retrotransposons, especially of the IAP family, in the MORC1-deficient testis (Supplementary Fig. 5a–c), similar to that observed in *Dnmt3l*^*−/−*^19. These LTR transposon-derived piRNAs corresponded to primary sense piRNAs (Supplementary Fig. 5d,e), suggesting that they are probably more abundant simply because the underlying mRNA species are derepressed in *Morc1*^*tg/tg*^, and some fraction are converted to piRNAs (Supplementary Fig. 5f). Hence, our results indicate that MORC1 acts in a transposon-silencing pathway independent of piRNA production.

### Hypomethylation of transposable elements in *Morc1* ^*tg/tg*^

**Figure 3:**
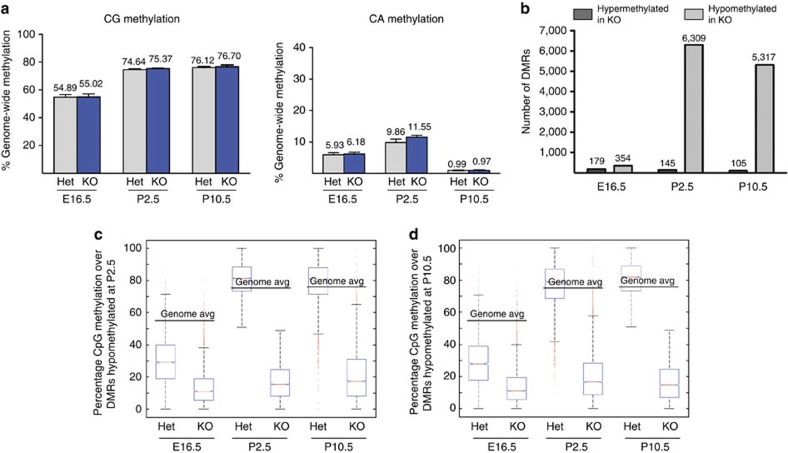
MORC1 regulates DNA methylation in a stage- and locus-specific manner. (**a**) Total CG and CA methylation levels in *Morc1*^*tg/+*^ and *Morc1*^*tg/tg*^ sorted germ cells. Three to four replicates per genotype and time point were analysed; mean and s.e. are indicated. (**b**) Number of hypermethylated and hypomethylated DMRs, calculated using pooled BS-seq data at each time point. (**c**) Boxplots of DNA methylation levels at E16.5, P2.5 and P10.5 are shown for the set of regions that were computed as hypomethylated at P2.5 in *Morc1*^*tg/tg*^ germ cells. (**d**) Box plots of levels of DNA methylation levels in germ cells at E16.5, P2.5 and P10.5 are shown for the set of regions that were computed as hypomethylated at P10.5 in *Morc1*^*tg/tg*^. For **c**,**d**, each DMR constitutes one point in each box plot. Red lines, median; edges of boxes, 75th (top) and 25th (bottom) percentiles; whiskers, minimum and maximum points within 1.5 × the interquartile range; red dots, outliers.

Because of the resemblance between transposons derepressed in *Morc1*^*tg/tg*^ and *Dnmt3l*^*−/−*^ testes (Fig. 2f,g), we sought to examine whether Morc1 might affect global DNA methylation levels. To address this, we performed whole genome bisulfite sequencing at E16.5, P2.5 and P10.5 on sorted *Morc1*^*tg/tg*^ and control germ cells isolated as above. At E16.5, the germline is undergoing *de novo* DNA methylation and by P2.5 *de novo* methylation is largely complete. Between roughly P2.5 and P10.5, germline cells re-enter the cell cycle and either initiate the first wave of spermatogenesis to generate meiotic cells or localize to the basement membrane and generate the long-term self-renewing spermatogonial stem cell population^20^,^21^. In contrast to *Dnmt3l*^*−/−*^ mutant germ cells that show a dramatic global reduction in DNA methylation^22^, we found no change in global levels of methylation at any time point in *Morc1*^*tg/tg*^-sorted germ cells (Fig. 3a). Thus, despite the similar morphological phenotypes and transposon expression defects of *Morc1*^*tg/tg*^ and *Dnmt3l*^*−/−*^ mice, MORC1 does not act by controlling *de novo* or maintenance methylation at a genome-wide level.

In mammals, DNA methylation is very dynamic and promoter DNA methylation frequently correlates with gene repression. To determine whether there may be localized defects in DNA methylation in *Morc1*^*tg/tg*^, and whether these are associated with derepressed transposons identified by RNA-Seq, we calculated statistically significant differentially methylated regions (DMRs) in the *Morc1*^*tg/tg*^ germ cells relative to the *Morc1*^*tg/+*^ control. At E16.5, we found very few DMRs (Fig. 3b). However, at P2.5 we identified 6,309 hypomethylated regions (Supplementary Data 3) but only 145 hypermethylated regions (Supplementary Data 4), indicating that *Morc1*^*tg/tg*^ germ cells have locus and stage-specific DNA methylation defects (Fig. 3b). In addition, the overwhelming majority of regions identified as hypomethylated at P2.5 remain hypomethylated at P10.5 (Fig. 3c) and only a few regions lost methylation between P2.5 and P10.5 (Fig. 3d).

**Figure 4:**
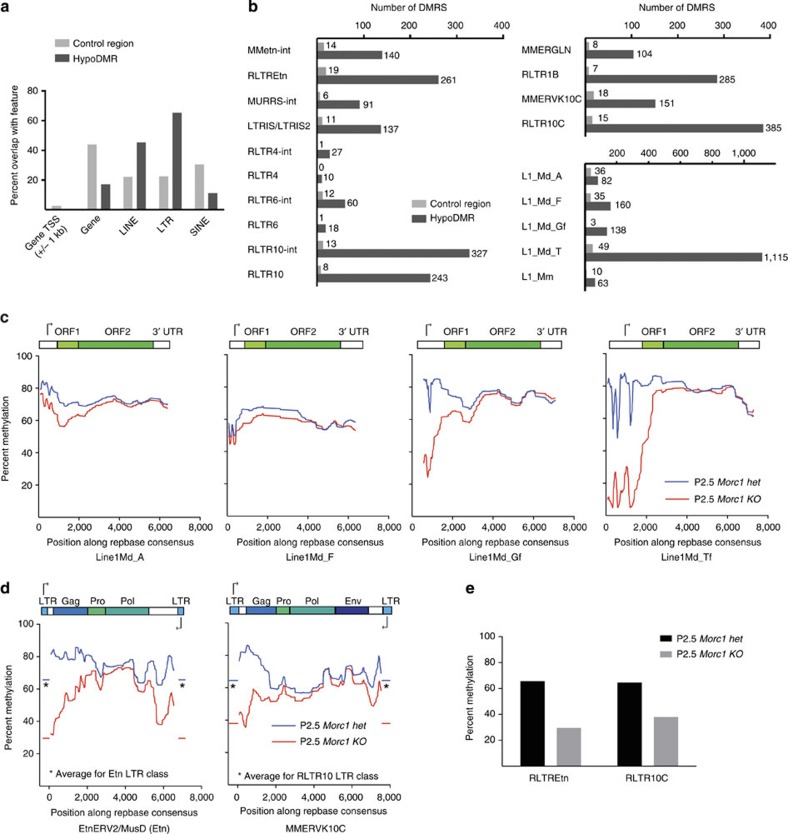
Hypomethylated regions in *Morc1*^*tg/tg*^ correspond to upregulated transposon classes. (**a**) The overlap of hypomethylated DMRs and control regions (randomly selected regions whose methylation is unaffected by loss of MORC1) with genes and transposon classes is indicated. A DMR or control is counted as overlapping with a feature if there was at least one basepair overlap. (**b**) Overlap of hypomethylated DMRs with transposon classes upregulated in *Morc1*^*tg/tg*^. Metaplot of methylation over LINE (**c**) and LTR (**d**) retrotransposons. BS-seq data were mapped to annotated RepBase consensus sequences for each transposon class and methylation is plotted at each CG in the annotated RepBase consensus sequence relative to the consensus sequence. The first 50 bases from each element, which often have low read coverage, are omitted. As it was usually not possible to determine the orientation of LTR-derived reads relative to LTR transposons, the average methylation level for the relevant LTR species is shown. (**e**) Global hypomethylation of LTR species corresponding to upregulated transposon classes. Again, BS-seq data were mapped to RepBase consensus sequence for these LTRs.

The hypomethylated DMRs in *Morc1*^*tg/tg*^ germ cells were highly enriched for LINE and LTR transposons rather than protein-coding genes compared with control regions (see Supplementary Methods), consistent with the transposon expression defects observed in *Morc1*^*tg/tg*^ germ cells (Fig. 4a). Indeed, 93.9% of hypomethylated DMRs contained an LTR or LINE, compared with 40.6% of control DMRs. The hypomethylated DMRs were strongly concentrated in the categories of transposons that showed evidence of derepression (Fig. 4b–d) during some stage of development before meiosis.

A partial exception to this trend were IAP elements. Hypomethylated DMRs were strongly enriched for IAP elements and corresponding LTRs (Supplementary Fig. 6a), but there was a poor correspondence between the extent to which a subcategory of IAPs was upregulated and the frequency of overlap with DMRs (Fig. 2d and Supplementary Fig. 6b). This is probably because certain highly similar repetitive elements such as LTR1 give very few uniquely mapping reads and are therefore missing from the data set. To overcome this, we also mapped the BS-seq data to RepBase consensus sequences for relevant transposons. We confirmed hypomethylation of the upregulated IAPLTR1 class (Supplementary Fig. 6c). Mapping to repeat consensus sequences also confirmed hypomethylation of LINE and LTR classes, which frequently overlap with DMRs (Fig. 4c–e).

Only 20 protein-coding genes contained an annotated transcription start site (TSS) within 1 kb of a hypomethylated DMR (Supplementary Data 5) and only 3 contained a TSS within a DMR. Interestingly, all three of these genes (*Nebulin*, *Tmc2* and *Cdkl4*) contain an RLTR10A transposable element immediately upstream of the TSS and all three genes showed a statistically significant increase in expression (Supplementary Fig. 7 and Supplementary Data 5). Thus, at a very few loci, MORC1 regulates genic expression, probably as a byproduct of its transposon repression activity in the local neighbourhood.

Considering MORC1’s role as local modulator of DNA methylation, we examined changes in methylation in the three well-characterized paternally methylated imprinted loci^23^. Methylation occurred normally at two of the three loci (*H19* and *Dlk1-Gtl2*), but the imprinting control region of *Rasgrf1* showed increased transcription and hypomethylation in the *Morc1* mutant (Supplementary Fig. 8). Interestingly, this is a transposon-rich area, which has previously been demonstrated as a target of the piRNA pathway^24^ (see Discussion below).

Of the very few hypermethylated loci observed in the *Morc1* mutant, most were not conserved across time points and are probably a consequence of biological or statistical noise. However, 15 hypermethylated DMRs were reproducible between P2.5 and P10.5. Nine of these 15 were embedded in 2 transcripts upregulated in *Morc1*^*tg/tg*^: 6 DMRs contained within the body of the *Cdkl4* gene described above and 3 DMRs in an unannotated transcript probably originating from a hypomethylated IAPLTR1 element (Supplementary Fig. 9). These are probably examples of transcriptional run-through from a nearby promoter causing methylation of a locus, a phenomenon that has been described for some imprinted loci^25^.

### DMRs are sites of transposon transcriptional initiation

The highly localized affect of MORC1 on the germline epigenome suggests that MORC1 may function at the transcriptional start sites of transposons to facilitate their silencing and methylation. In support of this, we discovered that hypomethylation in *Morc1*^*tg/tg*^ mutant germline cells was concentrated at the 5′- ends of LINE elements coincident with the location of transcriptional initiation (Fig. 4c)^26^. Furthermore, LTR transposons, which are typically flanked by LTRs that serve promoter and enhancer functions^27^, showed hypomethylation on both ends in *Morc1*^*tg/tg*^ (Fig. 4d), and the LTRs themselves are heavily hypomethylated (Fig. 4d,e).

**Figure 5:**
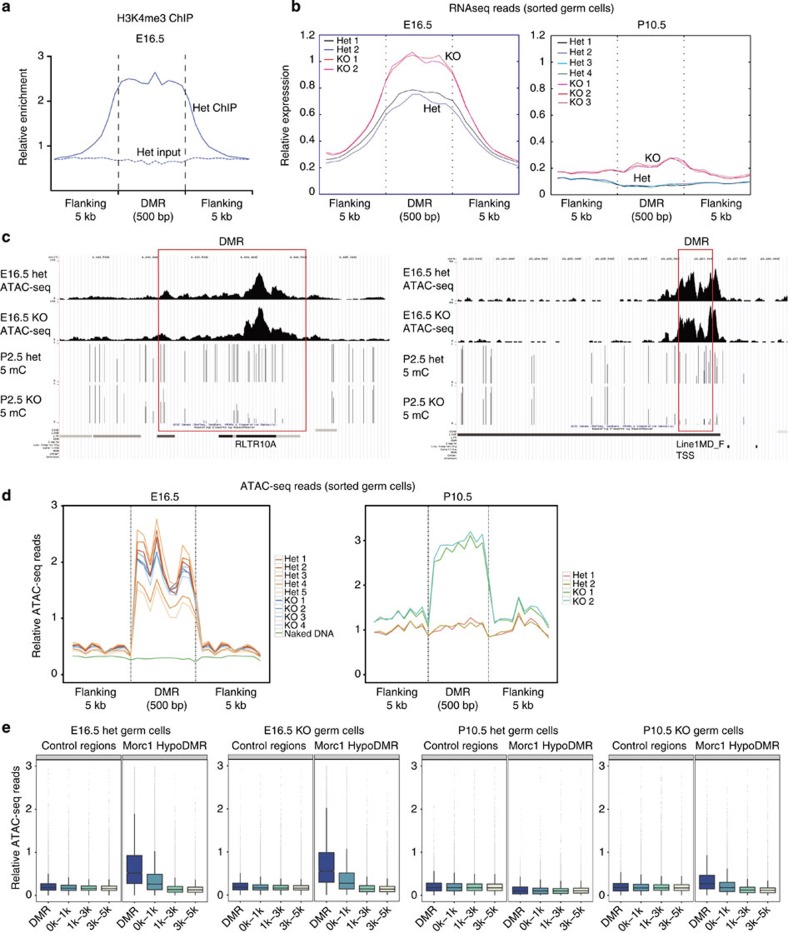
Hypomethylated regions in *Morc1*^*tg/tg*^ correspond to TSS of transposons active in late embryogenesis. (**a**) H3K4me3 abundance at E16.5 is calculated within regions identified as hypomethylated DMRs (Hypo DMR) in P2.5 *Morc1*^*tg/tg*^ germ cells. (**b**) Average distributions of uniquely mapping E16.5 RNA-Seq reads (left) and P10.5 RNA-seq reads (right) from individual replicates are plotted over regions identified as hypomethylated at P2.5. (**c**) Pooled E16.5 ATAC-seq reads are plotted relative to methylation distribution at two loci with hypomethylated DMRs. Each CG is represented as by a bar, with the height of the bar indicating the frequency with which the CG is methylated. A dot at a position indicates no methylation. At least one read must map to the CG for a bar to appear. (**d**) ATAC-seq reads from individual replicates at E16.5 (left) and P10.5 (right) are plotted relative to DMRs. (**e**) ATAC-seq read abundance at DMRs and adjacent regions is represented as a boxplot, with each DMR constituting one point in the boxplot. For **a**–**e**, DMRs refer to regions hypomethylated in *Morc1^tg/tg^* germ cells at P2.5.

We also noted that hypomethylated DMRs in *Morc1*^*tg/tg*^ germ cells were late targets for *de novo* methylation during the course of epigenetic reprogramming, since in control *Morc1*^*tg/+*^ cells these genomic regions were also hypomethylated relative to the genome average at E16.5 (Fig. 3c,d and Supplementary Fig. 10). This suggests that these loci are somewhat resistant to *de novo* methylation. Consistent with this possibility, we also discovered that these *Morc1* affected genomic regions have increased H3K4me3 relative to control regions of the genome in WT cells E13.5 (Supplementary Fig. 11a)^28^. To determine whether these loci are enriched in H3K4me3 during the dynamic *de novo* methylation of the germline genome, we performed chromatin immunoprecipitation (ChIP) analysis for H3K4me3 at E16.5 and confirmed that these regions still exhibited higher H3K4me3 (Fig. 5a). In contrast, other chromatin marks such as H2B10ac, K3K27ac and H3K27me3 showed no correlation with *Morc1*^*tg/tg*^ DMRs (Supplementary Fig. 11b). It is well established that H3K4 methylation antagonizes *de novo* DNA methylation by blocking DNMT3A/3L binding to histone H3 (ref. 29). Thus, the presence of H3K4me3 could potentially explain why these loci methylate with slow kinetics and require an additional factor (MORC1) for eventual silencing and methylation. It is also well established that H3K4me3 is a mark of transcriptional start sites, consistent with the idea that many DMRs are TSSs for transposons that are active in the embryonic germline.

At E16.5, we found that RNA transcripts were significantly elevated at *Morc1*^*tg/tg*^-hypomethylated DMRs relative to the surrounding areas, even in control *Morc1*^*tg/+*^ germ cells (Fig. 5b). However, by P10.5, this RNA expression was severely repressed in control *Morc1*^*tg/+*^ germ cells with modest but increased expression in *Morc1*^*tg/tg*^ (Fig. 5b). This data is consistent with a model in which the hypomethylated DMRs correspond to TSSs that are normally methylated and suppressed during development.

To further confirm that these DMRs correspond to transcriptional start sites, we employed ATAC-seq, which can be used to identify areas of open chromatin that are a signature of promoter and enhancer sites^30^. We confirmed that ATAC-seq can be accurately adopted for small sample sizes, that reads cluster near transcriptional start sites in E16.5 germ cells, and that ATAC-seq read density at the TSS correlates with gene expression (Supplementary Fig. 12). We found that ATAC-seq peaks overlapped tightly with DMRs (Fig. 5c,d) and most DMRs showed ATAC-seq reads substantially elevated over background (Fig. 5e). In contrast, reads from the naked DNA control were not enriched over DMRs (Fig. 5d). At P10.5, a more limited subset of DMRs exhibited elevated ATAC-seq reads (Fig. 5e), consistent with the observation that transcription is retained only at some DMRs in postnatal germ cells (Supplementary Fig. 13a,b). Other sites lose ATAC peaks and transcription (Supplementary Fig. 13c), either because relevant transcription factors are absent or because other mechanisms of transposon silencing are effective. Importantly, at E16.5, where we observe expression from DMR regions in both *Morc1*^*tg/+*^ and *Morc1*^*tg/tg*^ cells, we also observed a high ATAC-seq signal in both *Morc1*^*tg/+*^ and *Morc1*^*tg/tg*^ cells (Fig. 5d). In contrast, at P10.5, where DMRs are silenced in heterozygotes but remain expressed in *Morc1*^*tg/tg*^, we only observed high ATAC-seq signal in the *Morc1*^*tg/tg*^ cells (Fig. 5d). These results support the view that DMRs in *Morc1*^*tg/tg*^ cells correspond to promoters of transposons that fail to silence properly, leading to an inappropriately open chromatin state, and ectopic transposon expression, which is retained at P10.5, even after MORC1 expression has ceased.

## Discussion

The results of this study identify MORC1 as a critical regulator of transposon repression in the male germline. MORC1 does not act as a global regulator of DNA methylation. Instead, MORC1 functions to facilitate DNA methylation of a variety of transposons in the germline with very little effect on the expression or methylation of protein-coding genes. The observation that *Morc* homologues are required for gene silencing in *Arabidopsis*, *C. elegans* and now mammals suggests that the *Morc* family of proteins constitute conserved epigenetic regulators that probably function in a wide variety of eukaryotic organisms and developmental contexts.

The *Morc1*^*tg/tg*^ phenotype of transposon derepression and a block in meiosis prophase I superficially resembles the phenotype observed in mice deficient for proteins involved in the pre-pachytene piRNA pathway, including *Mili*^31^,^32^, *Miwi2* (ref. 12), *MitoPLD*^33^, *Mov10L1* (refs 34, 35), *Mael*^36^,^37^, *Tdrkh*^38^, *Tdrd9* (ref. 13) and *MVH*^39^,^40^. What distinguishes *Morc1*^*tg/tg*^ from these characterized pre-pachytene piRNA mutants, however, is the apparently normal piRNA biogenesis in *Morc1*^*tg/tg*^ (Table 1). We do note similarities in the pattern of hypomethylation in *Morc1*^*tg/tg*^ and *Mili*^*−/−*^ mutant germ cells, including the *Rasgrf1* imprinting control region^24^, as well as many of the same transposon families^41^. The dissimilarity in transposon repression observed in *Miwi2*^*−/−*^ and *Morc1*^*tg/tg*^ germ cells (Fig. 2f,h) suggests that MORC1’s role in the nucleus is independent from the nuclear piRNA pathway mediated by MIWI2. It is possible that MORC1 participates downstream of the nuclear piRNA pathway during embryogenesis and has a separate, piRNA-independent silencing role during the postnatal stages. This could cause *Morc1*^*tg/tg*^ to have a broader transposon derepression phenotype than *Miwi2*^*−/−*^. Alternatively, there may exist a MILI-dependent, MIWI2-independent mechanism for promoting methylation of target loci.

TEX19.1 has also been implicated in transposon repression in the male germline and has no known link to the piRNA pathway^42^. However, TEX19.1 is cytoplasmic^42^,^43^, shows dysregulation only of MMERVK10C elements^42^,^44^ and *Tex19.1*^*−/−*^ has an incomplete infertility defect^42^,^43^. Thus, the *Morc1*^*tg/tg*^ and *Tex19.1*^*−/−*^ defects are fairly dissimilar and there is no evidence that they participate in the same pathway.

Although we have revealed a critical role for MORC1 in transposon silencing, the actual mechanism by which MORC1 promotes DNA methylation in the male germline is unknown. Our study suggests at least three potential routes by which MORC1 represses transposons and facilitates DNA methylation. One possibility is that MORC1 directly silences transcription, perhaps using its ATPase activity to compact chromatin, thereby reducing H3K4 methylation levels at target sites. This silencing would allow for normal *de novo* methylation by DNMT3L. A second possibility is that MORC1 could recruit an H3K4 demethylase, which would similarly promote DNA methylation. Either mechanism agrees with our observation that MORC1-hypomethylated DMRs originate from loci with increased H3K4me3 at E13.5. A third non-mutually exclusive possibility is that MORC1 directly recruits the DNA methylation machinery to target loci, mediating methylation and silencing.

In conclusion, a robust genome defense system in the male germline is critical to safeguard genome integrity. We have identified a new participant that acts by facilitating DNA methylation of specific repetitive elements classes.

## Methods

### Mice

FVB/N-*Morc*^tg/+(Tyr)1Az^/J mice (*Morc1*^tg^) were recovered from cryopreservation at the Jackson Laboratory and maintained by intercrossing brothers and sisters in the FVB/N background. Male *Morc1*^tg/tg^ mice were viable healthy but infertile, whereas female *Morc1*^tg/tg^ mice were viable healthy and fertile. For PCR genotyping, the WT allele was detected as a 347-bp band with the following primers; forward: 5′- ATGCAACTTGAGGGGAAACA -3′ and reverse: 5′- GCAGGAGTTATGCGATGTCA -3′, and the mutant allele was detected as a 244-bp band with the following primers; forward: 5′- AGTTAGCCGTTATTAGTGGAGAGG -3′ and reverse: 5′- AGAAAGCCTGCCTCAAAACA -3′. PCR conditions involved ten cycles of 94 °C, 65 °C and 68 °C, followed by 28 cycles of 94 °C, 50 °C and 72 °C. For sorting germ cells from E16-5–P2.5, *Morc1*^tg /tg^ females were crossed into the *Oct4-IRES-Gfp* mixed background. For embryonic staging, timed pregnancies were established and the day a vaginal plug was identified was called embryonic day 0.5 (E0.5). For postnatal time points, the day a litter was first observed was referred to a postnatal day 0.5 (P0.5).

All animal experiments were approved by The UCLA Institutional Animal Care and Use Committee, also known as the Chancellor’s Animal Research Committee.

### Antibodies

Murine Morc1 coiled-coil domain (amino acids 788–950), expressed in and purified from bacteria, was provided by Jiamu Du and Dinshaw Patel (Sloan Kettering). Anti-Morc1 antibody was raised in rabbit in collaboration with Rockland Immunochemicals.

Anti-LINE Orf1p antibody was provided by Alex Bortvin (Carnegie Institution for Science) and anti-IAP Gaga antibody was provided by Bryan Cullen (Duke).

### Immunofluorescence

Whole testes were fixed with 4% paraformaldehyde, immobilized in paraffin and sectioned. After removal of paraffin, sections were stained at the following antibody concentrations: anti-LINE Orf1p (1:300), anti-IAP Gag (1:300), anti-MORC1 (1:100), anti-VASA (1:100, R&D Systems AF2030), stained with fluorescent secondary antibody and mounted with DAPI (4',6-diamidino-2-phenylindole). Slides were imaged by Confocal microscopy.

### Embryonic germ cell purification

Collection of embryonic testes were performed following institutional approval for appropriate care and use of laboratory animals. Pregnant females were euthanized using CO_2_ and the embryos removed from the womb and stored on a 10-cm dish filled with chilled 1 × PBS. Testicles were removed from the embryos, placed in an individual 15-ml falcon tube with 3 ml of 0.25% Trypsin, with 3 μl of DNAse I 1 Unit per 1 μl (Life Technologies). Testes were incubated for 15 min at 37 °C. After incubation, the cells were agitated into suspension gently by pipetting. The trypsin was then quenched using 5 ml DMEM/10% fetal bovine serum (Life Technologies). The cells were centrifuged at 278*g* for 5 min and resuspended in 500 μl FACS buffer (1 × PBS 1% BSA). 7-Aminoactinomycin D was added at a 1:50 dilution (BD Biosciences) and the cells strained through BD FACS tubes (Corning) before analysis. Green fluorescent protein-positive cells were sorted into Buffer RLT (Qiagen) or ATL (Qiagen) for RNA or DNA extraction, respectively.

### Postnatal germ cell purification

Pups were euthanized using Isoflurane. The testes were removed using tweezers, placed in a 1.5-ml centrifuge tube and chilled on ice. When all testes had been removed, each pair was placed in 1 ml of type IV collagenase (Invitrogen) in an ultra-low-attachment six-well plate (Corning). All extraneous tissue and the tunica were removed and the seminiferous tubules were teased apart. The samples were then incubated at 37 °C for 15 min and centrifuged for 5 min at 278*g*. Testes were then resuspended in 500 μl of 0.25% Trypsin (Life Technologies) and incubated for 5 min at 37 °C. After the incubation period, the testes were agitated gently into suspension by pipetting. Five hundred microlitres of DMEM/10% fetal bovine serum was added and the samples were centrifuged for 5 min at 200*g*.

For the P2.5 timepoints, green fluorescent protein-positive cells were sorted as with embryonic time points. To sort germ cells at P10.5, the cells were washed with 1 ml FACS buffer and then resuspended in 500 μl FACS buffer. Cells were then incubated with 1:160 EPCAM PE (Biolegend 118205) and 1:250 μl H2-K^q^ 647 (Biolegend 115106) on ice for 20 min in the dark, then centrifuged 5 min at 200*g* and resuspended in 500 μl FACS buffer. DAPI was added (1:1,000, Life Technologies) and the cells were strained through BD FACS tubes (Corning) before analysis. SSC^lo^ EpCAM^hi^ H2-K^q−^ cells were sorted into Buffer RLT or ATL for RNA or DNA extraction, respectively.

### qRT–PCR of Morc1

For embryonic samples, gonads were pooled from approximately five to seven mice per time point. RNA was extracted by the TRIzol method and DNase-treated (Qiagen) before complementary DNA conversion (Superscript III, Life Technologies). Quantitative amplification of cDNA was performed in triplicate using SYBR Green quantitation (PCR primers listed below) on a 7900 HT Fast Real Time PCR System (Applied Biosystems).

*Rrm2*, F: 5′- CCGAGCTGGAAAGTAAAGCG -3′

R: 5′- ATGGGAAAGACAACGAAGCG -3′

Morc Exon 7, F: 5′- GACCCGCAGAAGTTCTTCA -3′

R: 5′- TGCTGCATCAATTCAGCTTC -3′.

### RNA preparation

RNA for was extracted from whole testes or cells using the RNeasy Micro Kit (Qiagen 74004). The material was quantified using a Nanodrop ND-1000 (Nanodrop) for RNA from whole testis or the Qubit RNA High Sensitivity Assay (Life Technologies) for RNA from sorted germ cells. RNA quality for material from whole testis was assessed by gel electrophoresis and visualization of the 28S and 18S rRNA bands.

### DNA preparation

DNA for bisulfite sequencing was extracted using the QiaAMP DNA Micro kit (Qiagen) and quantified using the Qubit dsDNA High Sensitivity Kit (Life Technologies).

### qRT–PCR of retrotransposons

qRT–PCR for retrotransposons was conducted using published primer sets^45^. One microgram total RNA was treated with DNAse I Amplification Grade (Life Technologies) and converted to cDNA using SuperScript II Reverse transcriptase and random hexamers as primer (Life Technologies). The samples were digested with RNAse H in accordance with manufacturer’s protocol. RT–PCR was then performed using iQ SYBR Green Mastermix (BioRad) with 750 nM concentration of each primer. The samples was amplified (PCR programme: 95 °C 10:00, 50x (95 °C 30s, 55 °C 30s, 72 °C 30s)) with detection of PCR product after each elongation step and determination of melting temperature after the completion of PCR. The reaction was performed using an Agilent Technologies Mx3005p qPCR System (Stratagene). Upregulation of transposon transcript in the mutant is estimated using difference of squares with glyceraldehyde 3-phosphate dehydrogenase as a control.

### RNA-seq library preparation

RNA from whole testes was processed for sequencing using a TruSeq RNA Sample Preparation Kit v2 (Illumina) with 250 ng–2 μg total RNA as starting material. Mutant and controls were always matched for starting RNA content. RNA from sorted germ cell was processed using the Ovation Human FFPE RNA-Seq Multiple kit (Nugen) using custom primers for depletion of murine rRNA provided by the manufacturer, using 10 ng of total RNA. Each library was prepared using RNA from one individual mouse.

### Small-RNA isolation and library preparation

Total RNA was isolated from embryonic testes using Ribozol. Thirty micrograms of total RNA was loaded on 12% urea-polyacrylamide (PAA) gel. The 19–30 nt fraction was excised and snap-frozen in liquid nitrogen in 400 μl 0.4 M NaCl. RNA was eluted from the gel overnight at 16 °C while shaking at 1,000 r.p.m. and then precipitated with 3 vol absolute ethanol. Pre-adenylated 3′-linker (/5′Phos/ TGGAATTCTCGGGTGCCAAGGAACTC /3′ddC/; 5′-DNA adenylation kit, NEB) was ligated to RNA overnight at 4 °C using Truncated RNA Ligase 2 (NEB). Ligation reactions were loaded onto 10% urea-PAGE, the 45–56 nt fraction was excised and nucleic acids extracted as above. 5′-Linker (5′- rGrUrUrCrArGrArGrUrUrCrUrArCrArGrUrCrCrGrArCrGrArUrC -3′) was ligated to the samples using RNA Ligase 1 (NEB) overnight at 4 °C. Ligation reactions were loaded on 10% Urea-PAA gel, 72–83 nt fraction was excised and nucleic acids extracted as above. Extracted samples were reverse transcribed (primer sequence: 5′- GGAGTTCCTTGGCACCCGAGA -3′) and library amplified by PCR using standard Illumina primers. Final libraries were excised from the agarose gel and sequenced.

### Bisulfite library preparation

Libraries were prepared using the Ovation Ultralow Methyl-Seq Library System (Nugen). Five to 25 ng DNA was used as starting material. Matched mutant and control samples always contained identical quantities of DNA. Unmethylated Lambda phage DNA (NEB) was spiked in at 0.5% input DNA quantity to determine conversion efficiency, which was consistently >98%. Each library was prepared using DNA from one individual mouse.

### ChIP sequencing

The ChIP sequencing (ChIP-seq) protocol was adapted from published sources^28^. FACS-sorted cells from an individual mouse were diluted to 292 μl with 1 × PBS at room temperature. Formaldehyde (Sigma) was added to a final concentration of 1% and the sample was incubated for 10 min at room temperature with rocking. One molar glycine was then added to yield a final concentration of 0.14 M and the samples were quenched 30 min with rocking. Cells were then spun at 425*g* for 10 min at room temperature. The cell pellet was flash frozen.

After thawing, the cells were resuspended in 200 μl lysis buffer (50 mM Tris-Cl pH 8.0, 20 mM EDTA pH 8.0, 1% SDS, 1 × Complete Protease Inhibitor (Roche)) and incubated on ice for 10 min. Samples were then subjected to a 9-min disruption using a Bioruptor on ‘High’ setting, with 30 s/30 s off disruption (hence, 4.5 min of disruption in total). Samples were spun at 14,000*g* for 10 min, to remove insoluble material. The soluble sample was diluted to 500 μl with dilution buffer (16.7 mM Tris pH 8, 0.01% SDS, 1.1% TritonX-100, 1.2 mM EDTA, 167 mM NaCl) and 10% of material was saved as input. Sample was precleared with 30 μl Protein A Dynabeads (Life Technologies) and preincubated for 1 h. The cleared material was incubated with 1 μl anti-H3K4me3 antibody (Millipore 04–745) overnight.

The samples were incubated with 30 μl Protein A Dynabeads and the precipitated material was recovered with a magnet. The beads were washed 2 × for 4 min with Buffer A (50 mM HEPES pH 7.9, 1% Triton X-100, 0.1% Deoxycholate, 1 mM EDTA, 140 mM NaCl), 2 × for 4 min with Buffer B (50 mM HEPES pH 7.9, 0.1% SDS, 1% Triton X-100, 0.1% Deoxycholate, 1 mM EDTA, 500 mM NaCl) and 2 × for 4 min with 10 mM Tris/1 mM EDTA. Bound material was eluted with 100 μl elution buffer (50 mM Tris pH 8.0, 1 mM EDTA, 1% SDS) at 65 °C for 10 min and then eluted a second time with 150 μl elution buffer.

The input samples were thawed and diluted with 200 μl buffer. Cross-linking of ChIP and input samples was reversed by incubating 16 h at 65 °C. Samples were cooled and treated with 1.5 μl of 10 mg ml^−1^ RNaseA (PureLink RNAse A, Invitrogen 12091-021) for 30 min at 37 °C. One hundred micrograms of Proteinase K was then added and the samples treated for 2 h at 56 °C. The samples were then purified using a Qiagen Minelute kit.

Samples were amplified by a SeqPlex DNA Amplification kit (Sigma) and then converted to libraries using an Ovation Rapid Library kit.

### ATAC-seq library construction

Libraries were generated using a method adapted from published protocol^30^. Briefly, FACS-collected cells from individual mice were spun at 500*g* for 5 min at 4 °C. Cells were resuspended in 50 μl lysis buffer (10 mM Tris pH 7.4, 10 mM NaCl, 3 mM MgCl_2_, 0.1% NP40, 1 × Complete Protease Inhibitor(Roche)) and spun at 500*g* for 10 min. at 4 °C to collect nuclei. The nuclei were resuspended in 50 μl Transposase reaction (25 μl 2 × Tagmentation buffer, 22.5 μl water, 2.5 μl Tn5 Transposase enzyme) and reacted for 30 min at 37 °C on a PCR machine. The material was purified using a Qiagen MinElute protocol, eluting with 14 μl EB (Qiagen).

To amplify ATAC-seq libraries from the treated material, we amplified using the Ad1 primer below and a different Ad2 primer for each sample, which functions as a barcode

Ad1: 5′- AATGATACGGCGACCACCGAGATCTACACTCGTCGGCAGCGTCAGATGTG -3′

Ad2.1_TAAGGCGA: 5′- CAAGCAGAAGACGGCATACGAGATTCGCCTTAGTCTCGTGGGCTCGGAGATGT -3′

Ad2.2_CGTACTAG: 5′- CAAGCAGAAGACGGCATACGAGATCTAGTACGGTCTCGTGGGCTCGGAGATGT -3′

Ad2.3_AGGCAGAA: 5′- CAAGCAGAAGACGGCATACGAGATTTCTGCCTGTCTCGTGGGCTCGGAGATGT -3′

Ad2.4_TCCTGAGC: 5′- CAAGCAGAAGACGGCATACGAGATGCTCAGG AGTCTCGTGGGCTCGGAGATGT -3′

Ad2.5_GGACTCCT: 5′- CAAGCAGAAGACGGCATACGAGATAGGAGTCC GTCTCGTGGGCTCGGAGATGT -3′

Ad2.6_TAGGCATG: 5′- CAAGCAGAAGACGGCATACGAGATCATGCCTAGTCTCGTGGGCTCGGAGATGT -3′

Ad2.7_CTCTCTAC: 5′- CAAGCAGAAGACGGCATACGAGATGTAGAGAGGTCTCGTGGGCTCGGAGATGT -3′

Ad2.8_CAGAGAGG: 5′- CAAGCAGAAGACGGCATACGAGATCCTCTCTGGTCTCGTGGGCTCGGAGATGT -3′

Ad2.9_GCTACGCT: 5′- CAAGCAGAAGACGGCATACGAGATAGCGTAGCGTCTCGTGGGCTCGGAGATGT -3′

Ad2.10_CGAGGCTG: 5′- CAAGCAGAAGACGGCATACGAGATCAGCCTCGGTCTCGTGGGCTCGGAGATGT -3′

The eluted material was amplified in 50 μl volume using 1.25 μM primer concentration and a 1 × concentration NEBNext High-Fidelity Master-Mix (NEB) (programme: 72 °C 5:00, 98 °C 30 s, 5 × (98 °C 10 s, 63 °C 30 s, 72 °C 1 min), 4 °C hold). After these five cycles of amplification, the tube was kept on ice.

A 5-μl aliquot was then removed and used to perform a 15-μl side reaction with identical concentrations of primer and enzyme as above, except that 0.6 × SYBR Green (Invitrogen S-7563) is included to monitor amplification. This side reaction was amplified on a Stratagene Mx3005p qPCR (Agilent) system with the following amplification conditions (98 °C 30 s, 20 × (98 °C 10 s, 63 °C 30 s, 72 °C 1:100)). The number of additional cycles ‘*N*’ required to reach one-fourth maximum fluorescence was observed. The purpose of this side reaction was to minimize the number of PCR cycles required used to generate the libraries, as length and GC bias increases with more amplification The remaining 45 μl of the reaction was then further amplified (98 °C 30 s, *N* × (98 °C 10 s, 63 °C 30 s, 72 °C 1:00), 4 °C) and the libraries were puried by a Qiagen MinElute kit eluting with 20 μl volume. Libraries were visualized by running on a 5% TBE gel and imaged by incubating for 20 min in 1 × SYBR Green/1 × TBE. Libraries quantified using the KAPA Library Quantification Kit (Kapa Biosystems).

### RNA-seq analysis

For all analyses, reads were trimmed to 50 bp and those mapping to ribosomal RNA (GenBank identifiers: 18S NR_003278.3, 28S NR_003279.1, 5S D14832.1, 5.8S K01367.1) by up to three mismatches were discarded.

*Analysis on repeat families*. Reads were then mapped to the mm9 genome allowing no mismatches and keeping reads that map up to 10,000 sites in the genome using Bowtie^46^. Each mapping read was assigned a score of 1/*n*, where *n* is the number of sites in the genome the read mapped to. Repeats were obtained from RepeatMasker. Expression values for each repeat family was calculated by adding the scores contained within the repeat body, divided by the total million reads mapped and average length (kb) of repeats within the family.

*Analysis on individual genes and repeats*. Reads were then mapped to known mm9 gene and repeat annotations by allowing up to two mismatches and only retaining reads that mapped to one location. When reads did not map to the annotated genes and repeats, the reads were mapped to the mm9 genome. Number of reads mapping to genes and repeats were determined by using HTSeq ( doi: 10.1101/002824) using default parameters. Expression values were calculated as reads per kilobase of exons per million mapping reads. Differential gene and repeat expression was determined by using DESeq^47^, by using default parameters.

### Whole-genome bisulfite sequencing

Reads were split into 50 bp reads before mapping. Reads were mapped to the mm9 genome as well as the lambda genome using BS seeker2 (ref. 48) using default parameters. Methylation levels were determined by #C/(#C+#T). For identifying DMRs, the genome was tiled into 500 bp bins and CG methylation levels in knockout and control were compared within each bin. Bins that had a methylation level difference of 50% as well as a false discovery rate<0.05 calculated by Fisher’s exact test corrected by the Benjamini–Hochberg procedure were selected. Finally, DMRs containing at least four cytosines in CG contexts, each covered by at least four reads were retained. Control regions were defined completely randomly, except that control regions have (1) exact same coverage of cytosines in CG contexts as the *Morc1*^*+/tg*^ data within DMRs; (2) WT CG methylation levels are similar as the *Morc1*^*+/tg*^ data within DMRs (<5%). (3) same number of regions per chromosome as DMRs. We defined genes as associated with DMRs when the TSS of an Ensembl transcript model was within 1 kb of a DMR.

To align to Repeat consensus sequences, the RepBase consensus sequences for 30 repetitive elements (B1_SINE, ERVB7 1-I MM (EtnERV2/MusD), IAP-d, IAPEY3_I, IAPEY_I, IAPEY_LTR, IAPEY3_LTR, IAPEZI, IAPLTR1_Mm, IAPLTR2_Mm, IAPLTR3, IAPLTR3_I, IAPLTR4, IAPLTR4-I, L1MdA_I, L1MdF_I, L1MdGf_I, L1MdTf_I, First 234 bases of GSAT_MM (Major_satellite), MMERGLN_I, MMERGLN_LTR, MERVL, MERVL_LTR, First 120 bases of SATMIN (Minor_satellite), MMERVK10C, RLTR10C, RLTR27_MM, RLTR6_MM, RLTR6I_MM, RLTRETN_MM and RSINE1 were combined into a microgenome. Then, whole-genome bisulfite sequencing reads were mapped to the microgenome using BSMAP^49^, accepting uniquely mapping reads only [−w 1], mapping to two forward possible strands [−n 0] and allowing 2 mismatches [−v 2]. Methylation levels were determined by #C/(#C+#T). The methylation levels at each CG site was calculated.

### Small RNA sequencing

Sequence adaptors were removed using a custom-designed dynamic programming algorithm that recognizes both exact and inexact matches, and the trimmed reads were aligned to the mm9 genome following a custom suffix array-based procedure^50^. Reads with lengths >24 nt were considered for piRNA analysis. Based on alignment coordinates, the reads were annotated as derived from exons, introns, transposons and other repeats according to the genome annotation obtained via UCSC Genome Bioinformatics^51^. Reads that had multiple valid alignments were annotated based on ten alignments selected at random, and the majority annotation was assigned as the final annotation. In case of ties, annotation was picked based on a fixed hierarchy principle^50^. Sense or antisense annotation was assigned to piRNA reads with respect to the strandedness of an underlying genomic feature. If a piRNA read contained U in position 1, such piRNA was considered as primary, while the presence of A in position 10 defined secondary piRNAs.

### ChIP-seq analysis

Previously published ChIP-seq data^28^ was obtained from GSE38165 in Gene Expression Omnibus. Reads were mapped to the mm9 genome using Bowtie by allowing up to two mismatches and only retaining reads that mapped to one location in the genome. Reads mapping to the same location were collapsed into one read. For all analyses, the data were normalized to total number of mapping reads in the library

### ATAC-seq analysis

Data were collected using 50 bp paired end sequencing on a HiSeq. In keeping with established methodologies^30^, reads were aligned to mm9 using Bowtie^52^ with the parameters –X2000 and –m1. The –X2000 parameter allows the fragments <2 kb to align and only unique aligning reads were collected (−m1). Duplicated reads were removed with samtools (rmdup function)^53^. Previous results show that for Tn5 transposase, the transposon binds as a dimer and insert two adaptors separated by 9 bp^54^. Thus, all reads aligned to the positive strands were offset by +4 bp and all reads aligned to the negative strands were offset by −5 bp.

## Additional information

**How to cite this article:** Pastor, W. A. *et al*. MORC1 represses transposable elements in the mouse male germline. *Nat. Commun.* 5:5795 doi: 10.1038/ncomms6795 (2014).

**Accession codes:** Raw sequencing data, including RNA-seq, whole-genome bisulfite-seq, small RNA-seq, ATAC-seq and ChIP-seq, generated for this study have been deposited in the GEO database under accession number GSE63048.

## Supplementary Material

Supplementary Information Supplementary Figures 1-13

Supplementary Data 1 Samples used in sequencing. A list of all samples used in sequencing as well as which figures use the data from that sequencing run

Supplementary Data 2 All repeats expression data. Expression of all annotated repetitive elements in all RNA-seq samples is listed. A full description of how these numbers are calculated is included in the Supplementary Methods. Briefly, these expression values are proportional to the number of reads mapping to a given repetitive element class divided by the total number of reads in that class.

Supplementary Data 3 Locations of hypomethylated DMRs in *Morc1^tg/tg^* germ cells in the mm9 mouse genome.

Supplementary Data 4 Locations of hypermethylated DMRs in *Morc1^tg/tg^* germ cells in the mm9 mouse genome

Supplementary Data 5 DMRs adjacent to genes.

## Figures and Tables

**Table 1:** piRNA abundance and characteristics in E16.5 *Morc1* ^*tg/+*^ and *Morc1* ^*tg/tg*^ testes.

	***Morc1*** **het**	***Morc1*** **KO**
Putative piRNA/miRNA	0.55	0.59
Sense/antisense	1.34	1.34
Primary/secondary	4.43	3.91

miRNA, micro RNA; piRNA, Piwi-interacting RNA; smRNA, small RNA.

Ratios of putative piRNA/miRNA, sense piRNA/antisense piRNA and primary piRNA/secondary piRNA populations are indicated for smRNA obtained from pooled E16.5 *Morc1*^*tg/+*^ and *Morc1*^*tg/tg*^ testes. No substantial defect in piRNA biogenesis is observed in MORC1-deficient testis.
