# Supplementary material for: MORC1 represses transposable elements in the mouse male germline
Source: Nat Commun. 2014 Dec 12;5:5795. doi: 10.1038/ncomms6795 (PMC4268658; doi:10.1038/ncomms6795)
Supplement: Supplementary Information — Supplementary Figures 1-13 [file ncomms6795-s1.pdf]

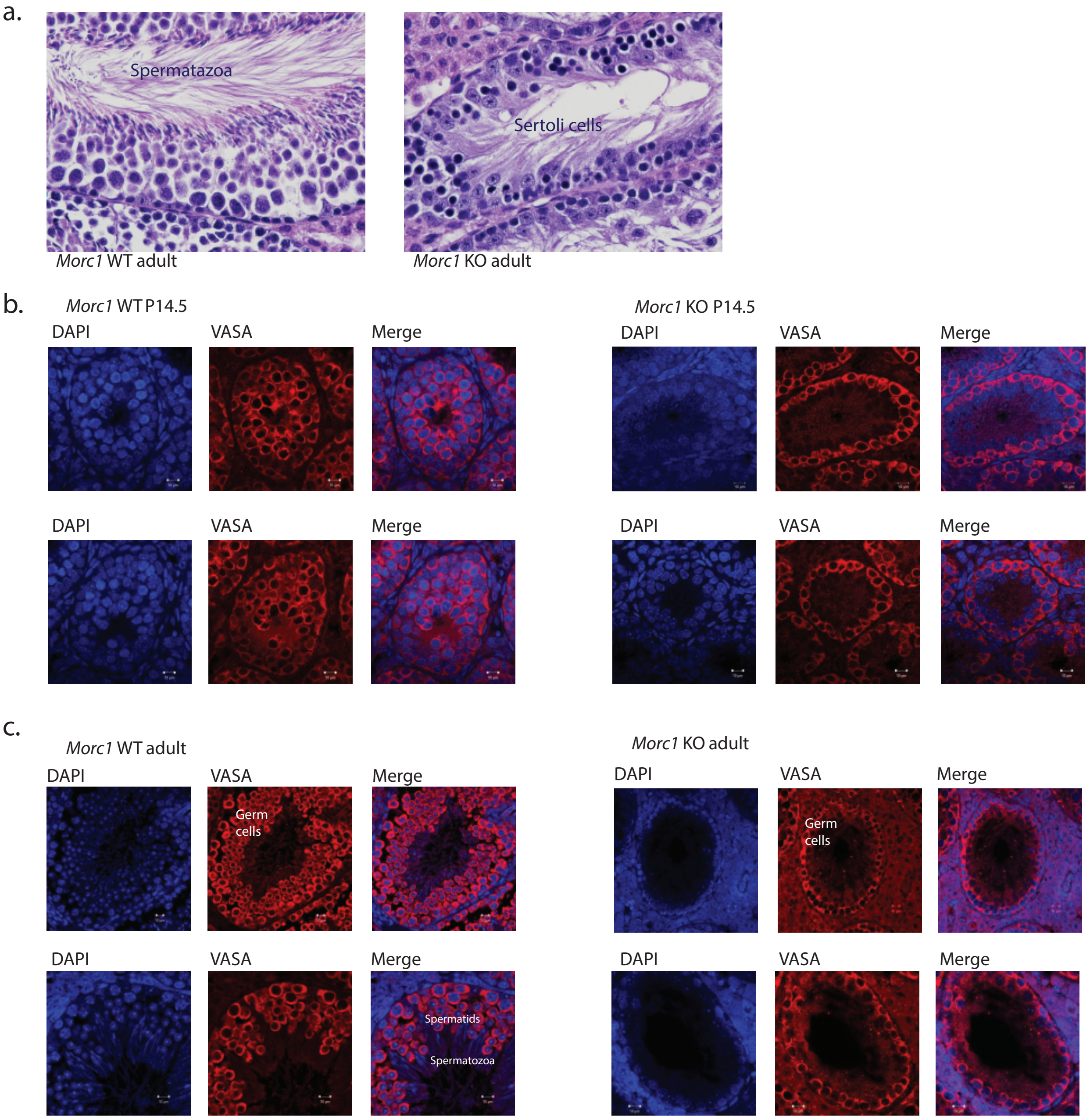

Supplementary Figure 1. Confirmation that *Morc1*$^{tg/tg}$ males are infertile. a, Histology of *Morc1*$^{+/+}$ and *Morc1*$^{tg/tg}$ adults. **b-c**, Detection of germ cells in *Morc1*$^{+/+}$ and *Morc1*$^{tg/tg}$ adults by immunofluorescence (IF). VASA is a marker of germ cell identity. Scale bars indicate 10µm.

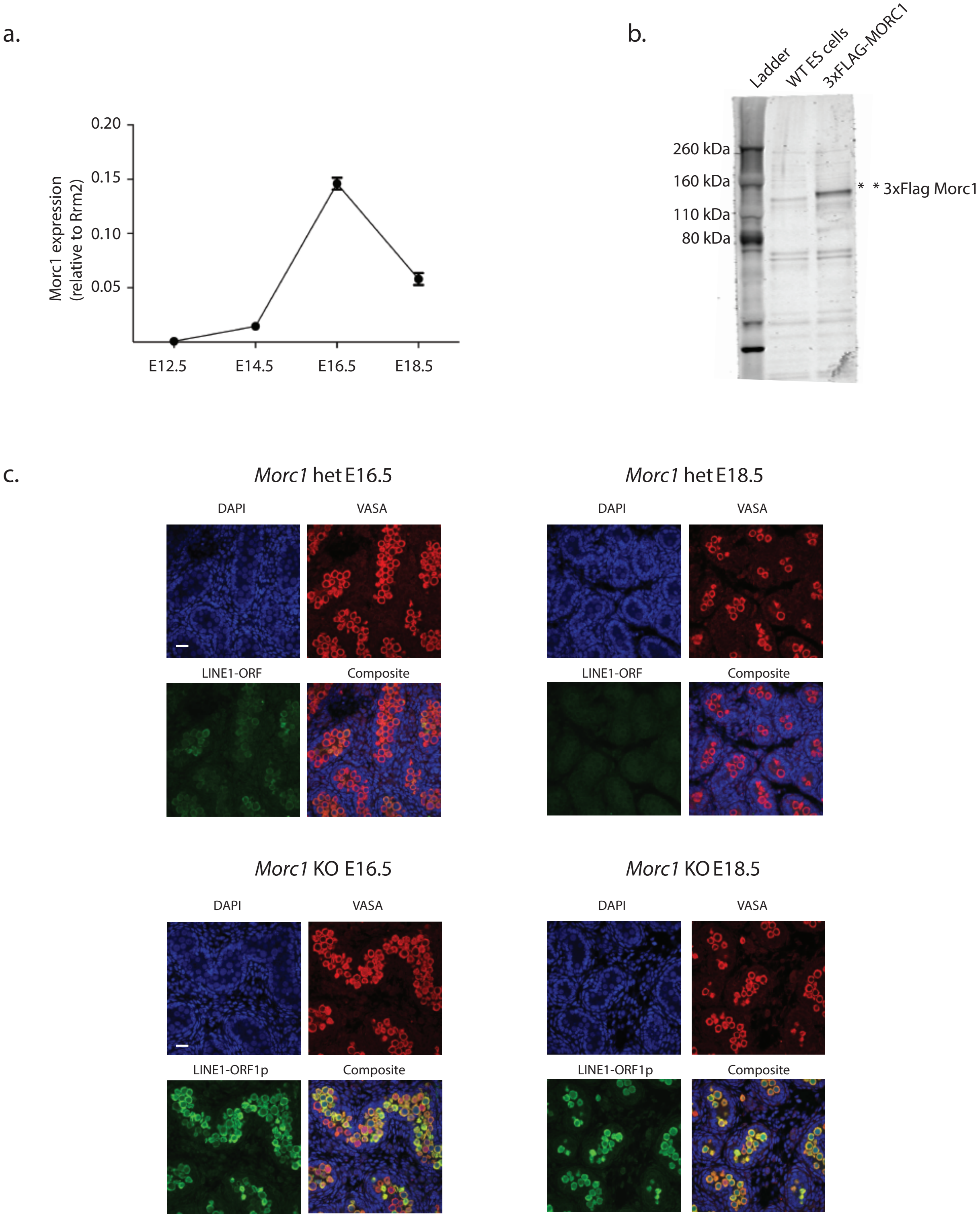

Supplementary Figure 2. Transposon derepression in *Morc1$^{tg/tg}$* arises concurrently with its expression in the germline. a, Relative *Morc1* mRNA expression in whole testis as measured by RT-PCR. Expression is shown relative to the housekeeping gene *Rrm2*. Six technical replicates per timepoint were analyzed, mean and standard error are plotted. **b**, Western blot confirming that anti-MORC1 coiled coil antibody recognize an overexpressed Flag-MORC1 in mouse embryonic stem cells. **c**, Immunoflourescent detection of LINE1-ORF1p protein in late embryogenesis (E16.5, E18.5). VASA is a marker ofgerm cell identity. Scale bars indicate 20μm.

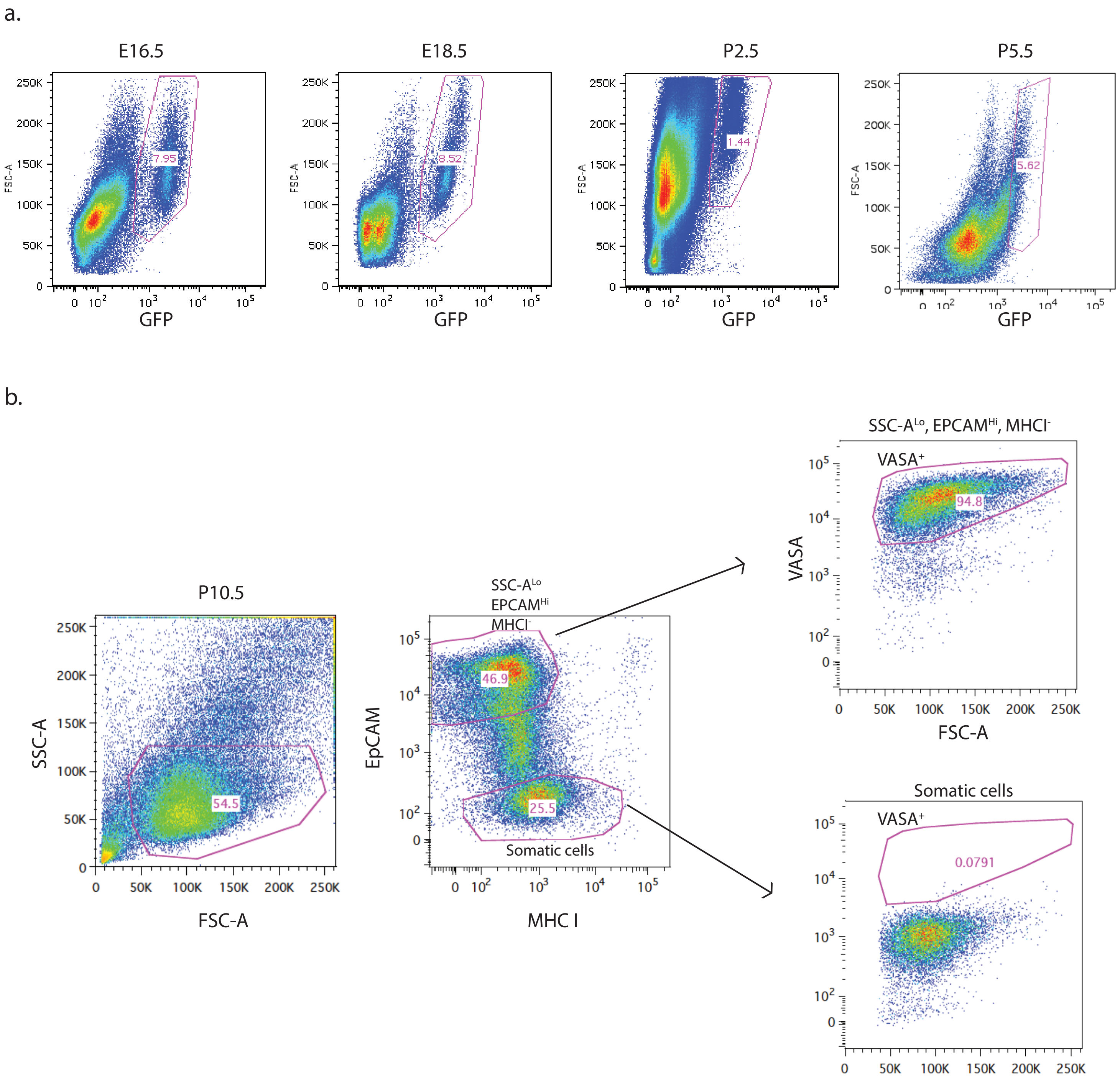

Supplementary Figure 3. Strategies for sorting pure germ cells. a, Flow cytometry plots showing a distinct eGFP$^+$ germ population in *Oct4-IRES-eGfp*. eGFP$^+$ cells could be used to sort embryonic and early postnatal germ cells effectively but was not useful after P2.5. **b,** Sorting a SSC-A^{Lo}, EPCAM^{Hi}, MHCI$^-$ population of cells from P10.5 whole testis, yields a 94.8% pure population of VASAHi (germ) cells. The EpCAM^{Lo} somatic cell population is shown as a control. VASA is an intracellular protein and thus is not an optimal marker for sorting cells for RNA-Seq applications. Therefore, the SSC-A^{Lo}, EPCAM^{Hi}, MHCI$^-$ population was used to sort P10.5 germ cells for whole genome-bisulfite sequencing and RNA-seq experiments.

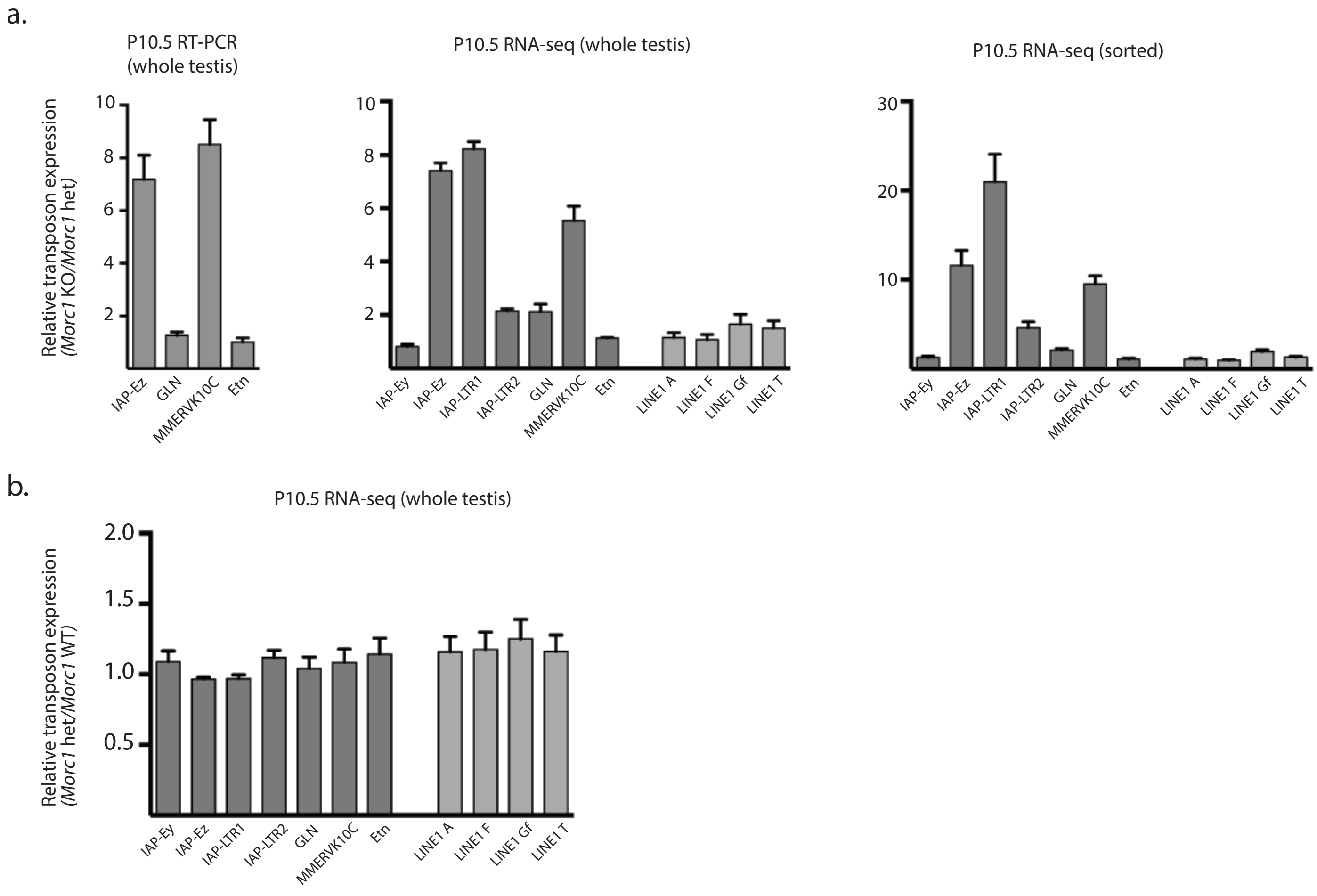

Supplementary Figure 4. Validation of sequencing data and strategy. a, RT-qPCR for select transposon classes at P10.5 in whole testis yields a similar pattern of upregulation in *Morc1*$^{tg/tg}$ as observed by RNA-Seq of whole testis and sorted germ cells, confirming the validity of each approach. The degree of upregulation is somewhat stronger in the sorted cells, probably because transcripts from the unaffected somatic cells blunt the increase in whole testis. b, There is no substantial increase in transposon expression in *Morc1*$^{tg/+}$ mice relative to *Morc1*$^{+/+}$, allowing us to use *Morc1*$^{tg/+}$ littermates of *Morc1*$^{tg/tg}$ mice as controls in subsequent experiments. 2-4 replicates per genotype were analyzed, mean and standard error are indicated.

a.

P10.5

	Het	KO
putative piRNA/miRNA	0.31	0.23
sense/antisense	7.15	8.93
primary/secondary	3.81	3.99

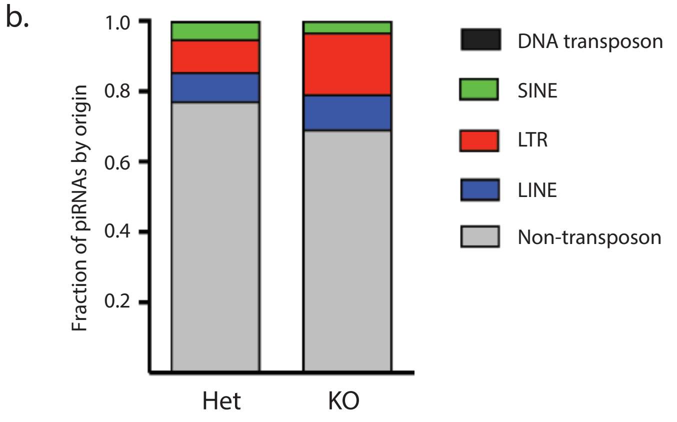

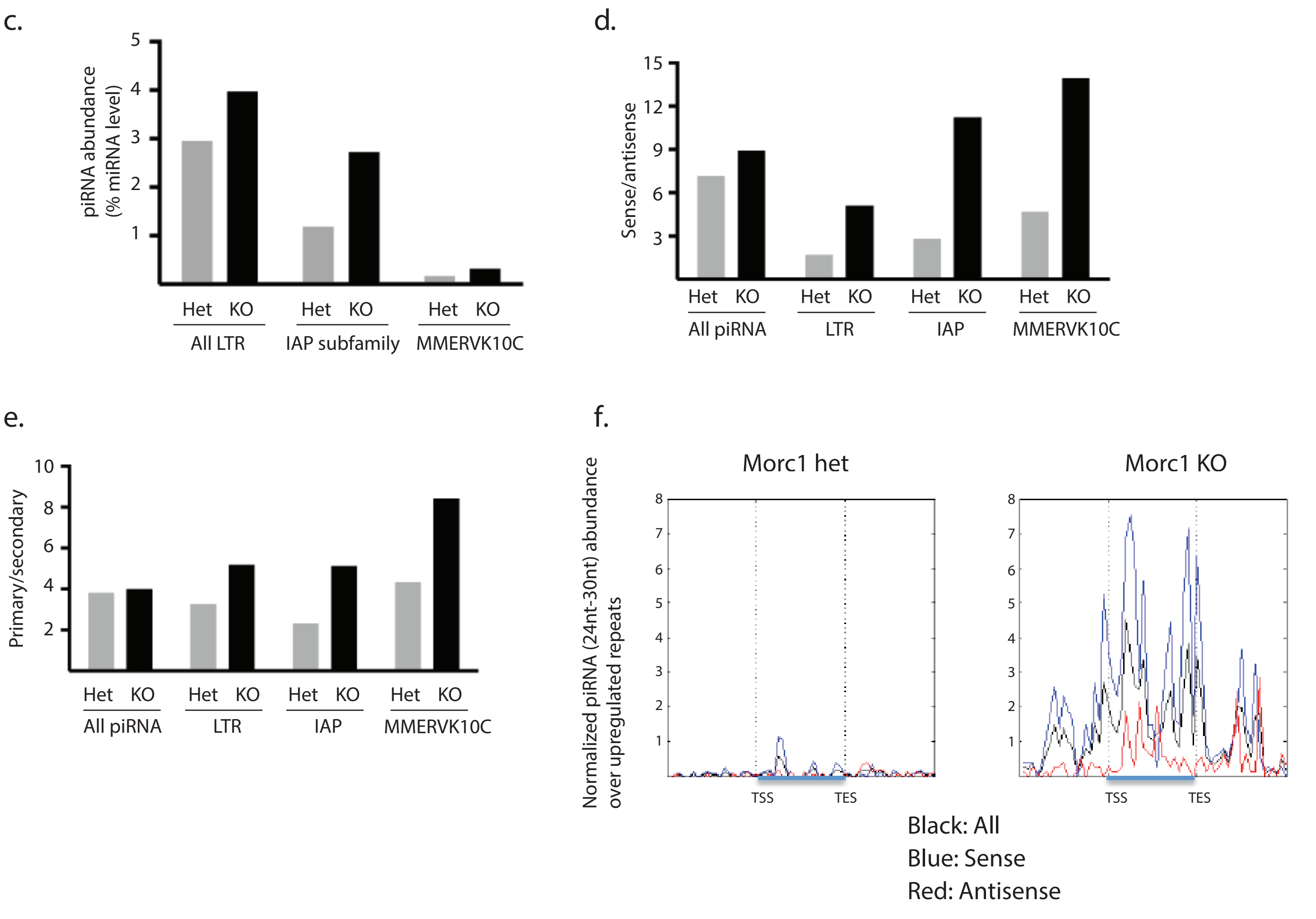

Supplementary Figure 5. Increased piRNA reads deriving from transposon elements upregulated at P10.5
a, Relative piRNA abundance and characteristics in P10.5 *Morc1*$^{tg/tg}$ and *Morc1*$^{tg/+}$ testes. **b**, piRNAs from P10.5 testes are plotted by transposon class. Consistent with overexpression of various LTR retrotransposons at this timepoint, a higher fraction of piRNA are derived from LTR retrotransposons. **c**, A large increase of piRNA derived from IAP and MMERVK10C in *Morc1*$^{tg/tg}$ is observed. **d**, The ratio of primary/secondary and **e**, sense/antisense piRNA of IAP derived sequence is plotted for *Morc1*$^{tg/+}$ and *Morc1*$^{tg/tg}$. An increase in primary, sense transcript is observed relative to other species, consistent with piRNAs being processed directly from transposons. **f**, Average distribution of sense and antisense piRNA over transposons upregulated in *Morc1*$^{tg/tg}$. Middle region represents the body of the transposon, and the flanking regions represent the 5′ (left) and 3′ (right) regions outside of the transposon. Both flanking regions were scaled to the same lengths as for the middle regions.

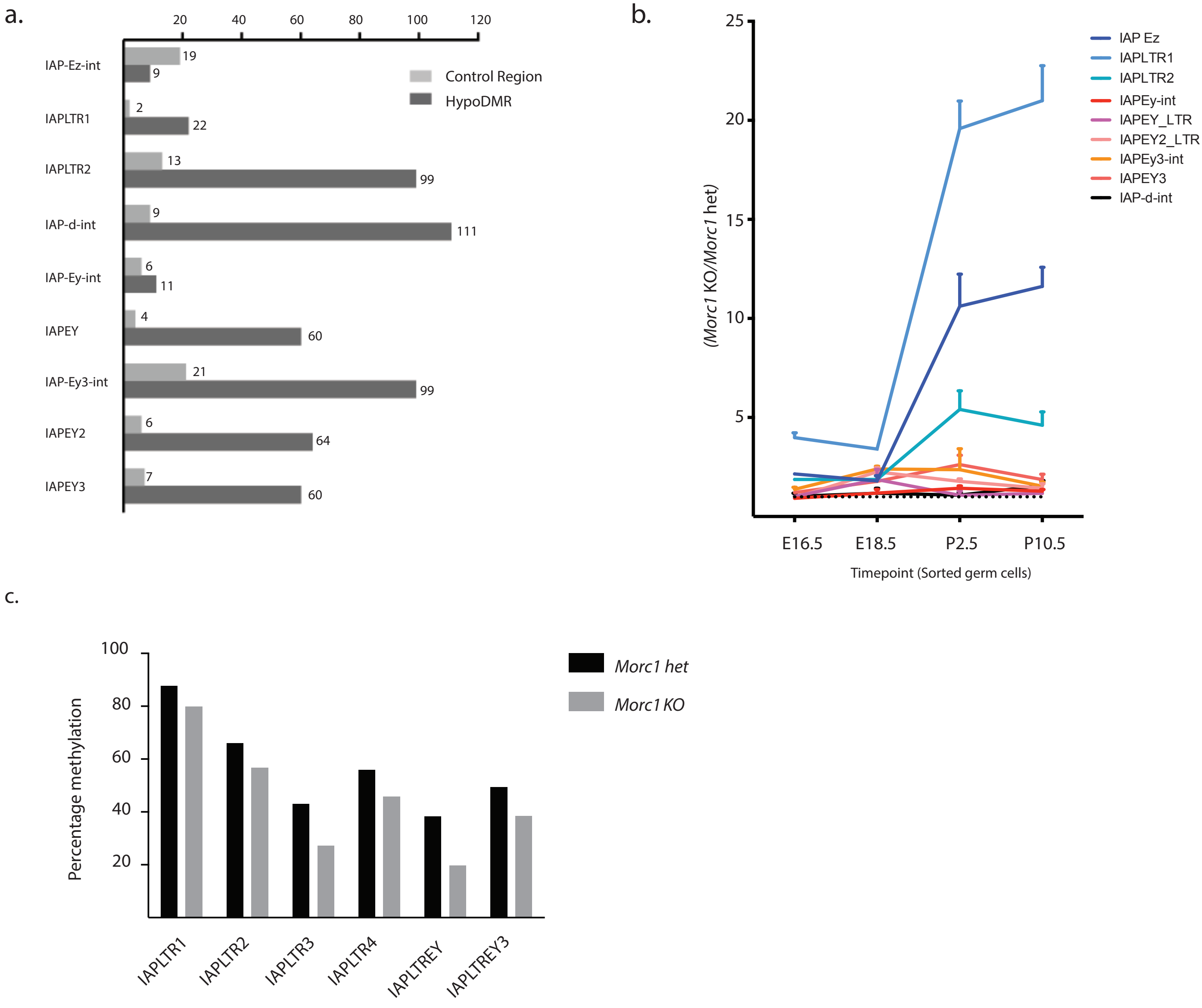

Supplementary Figure 6. Hypomethylation of IAP elements in *Morc1*$^{tg/tg}$. a-b, IAP elements show hypomethylation in the *Morc1*$^{tg/tg}$, however there is weak correspondence between the extent of upregulation (compare (**a**) to (**b**)) and frequency of hypomethylated DMRs. **c**, Overall, there is modest global hypomethylation of all IAP LTRs in *Morc1*$^{tg/tg}$. 2-4 replicates per genotype and timepoint were analyzed, mean and standard error are indicated.

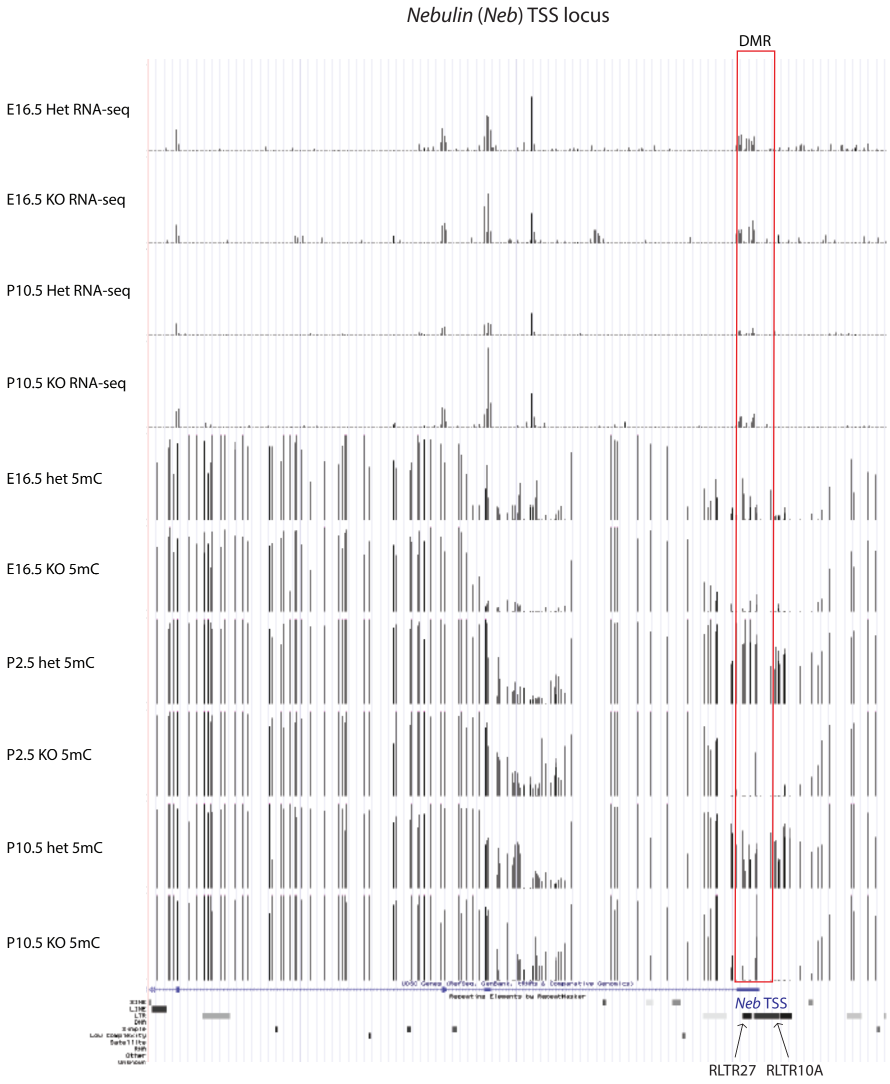

Supplementary Figure 7. Coordinated transposon and gene silencing at the *Nebulin* transcription start site. Pooled, uniquely mapping RNA-seq reads and BS-seq data is shown relative to the *Nebulin* locus. The TSS of the *Nebulin* gene is hypomethylated in *Morc1*$^{tg/tg}$, probably because of adjacent RLTR10A and RLTR27 LTRs. The *Nebulin* gene is correspondingly upregulated at P10.5. Each CG is represented as by a bar, with the height of the bar indicating the frequency with which the CG is methylated. A dot at a position indicates no methylation. At least one read must map to the CG for a bar to appear.

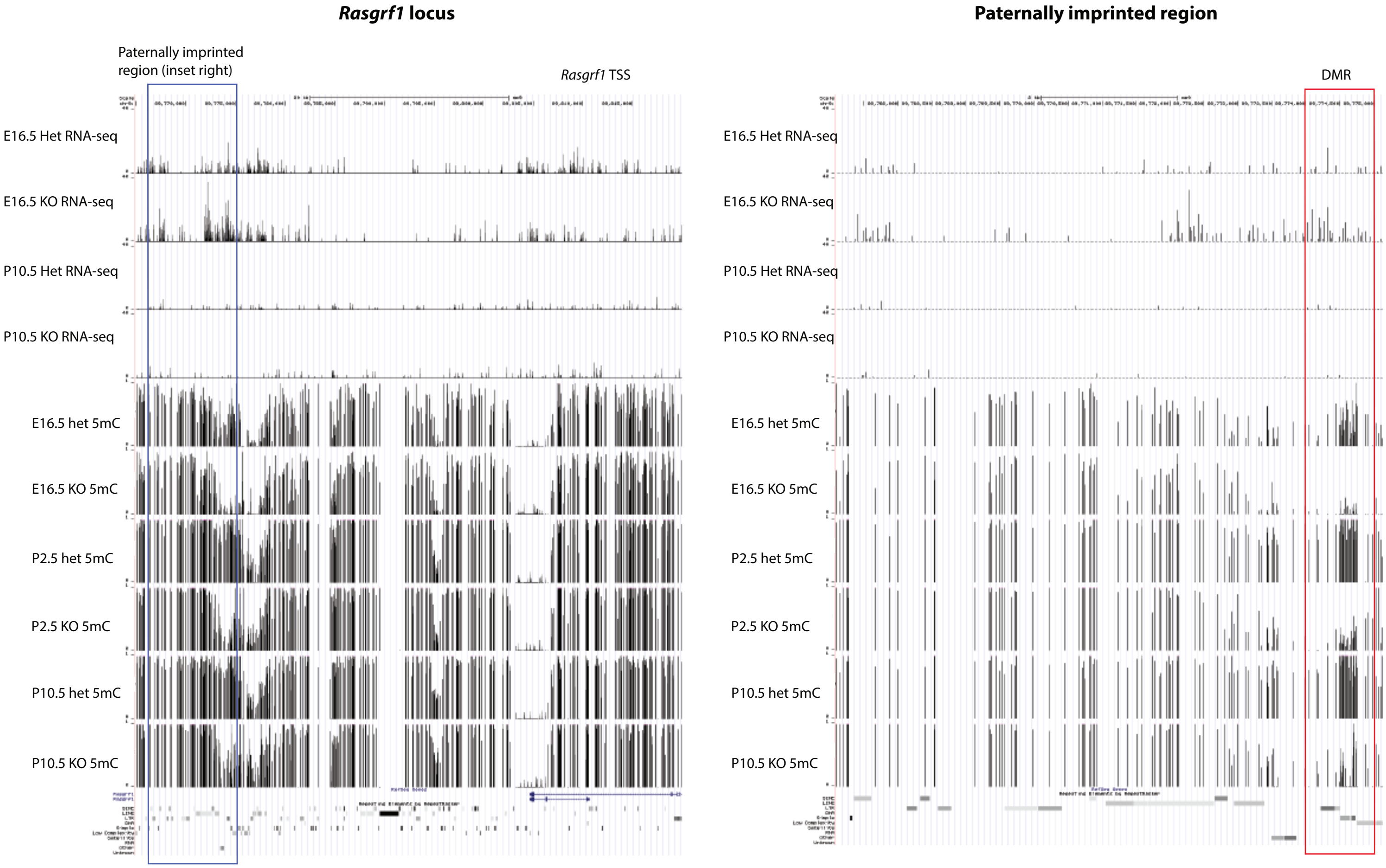

Supplementary Figure 8. Hypomethylation of the imprinting control region of the *Rasgrf1* locus. (*Left*) The paternally imprinted region of *Rasgrf1* is indicated relative to the transcription start site of the gene (*Right*), transcription and methylation over the paternally imprinted region is shown. Part of the paternally imprinted region is overexpressed and hypomethylated in the *Morc1*$^{tg/tg}$ mutant relative to the control.

a.

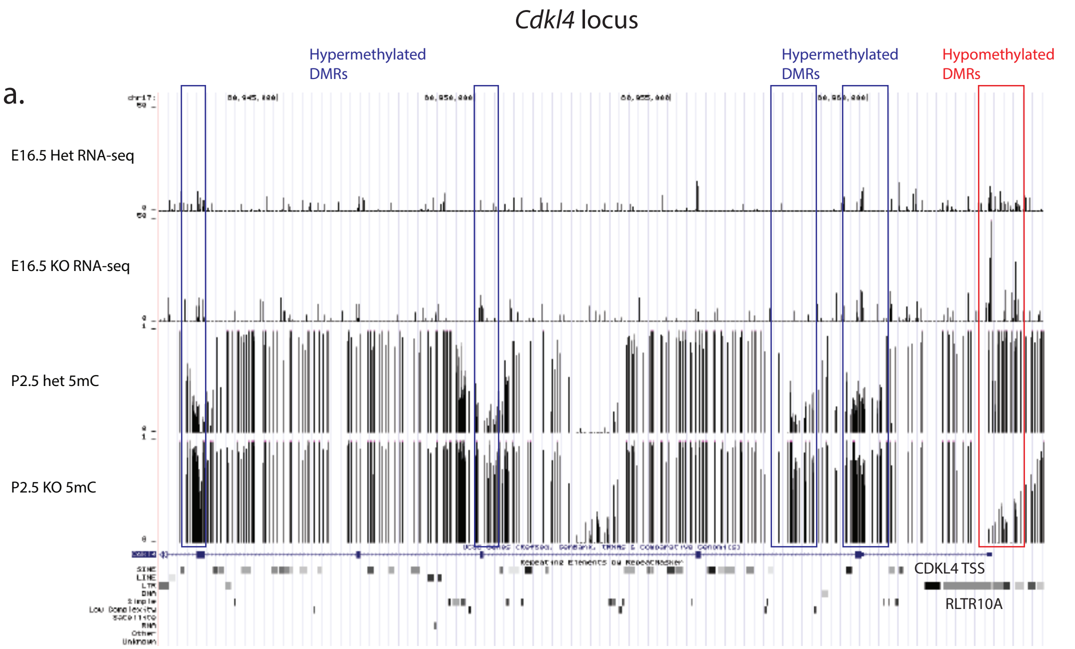

b.

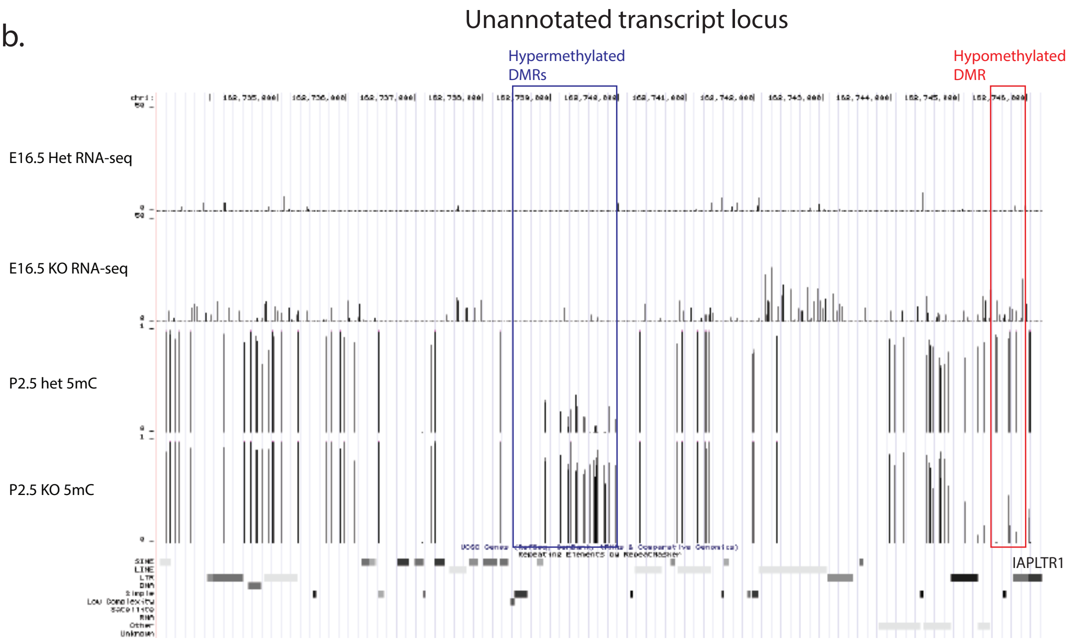

Supplementary Figure 9. Hypermethylation of select regions is linked to transcriptional readthrough. The *Cdkl4* (**a**) promoter and the probable promoter of an unannotated transcript (**b**) are hypomethylated in the *Morc1*$^{tg/tg}$. There is a corresponding increase in reads deriving from these transcripts and regions of hypermethylation develop in the transcript body.

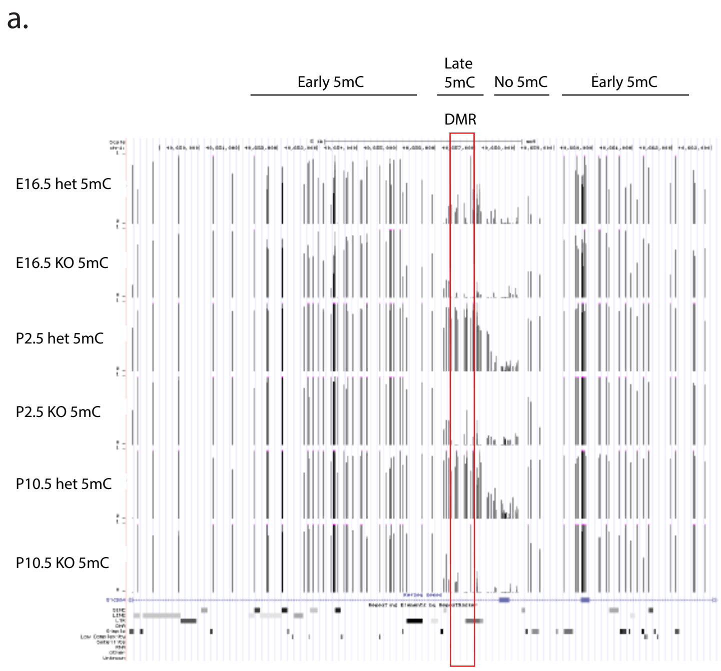

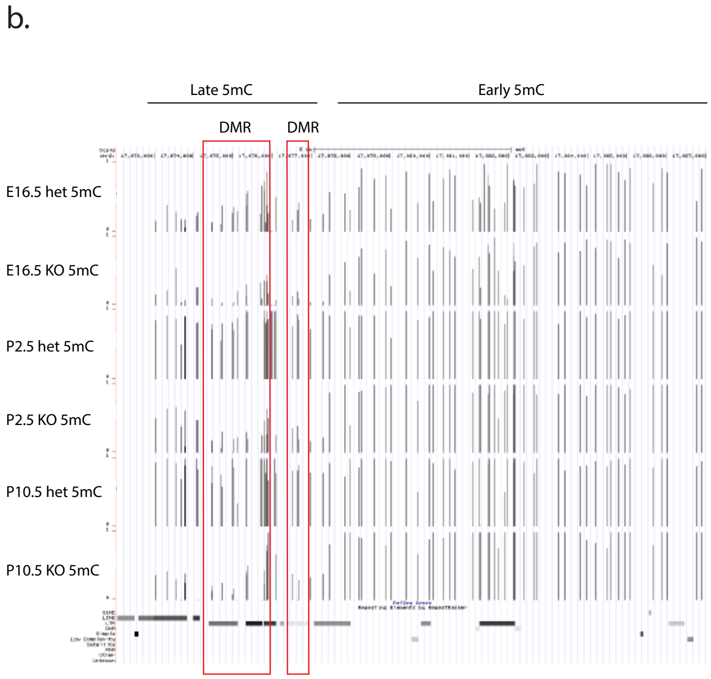

Supplementary Figure 10. Hypomethylated regions in *Morc1*$^{tg/tg}$ are methylated more slowly than other loci. Two regions (**a**) and (**b**) that show both "early" and "late" methylation are shown. Hypomethylated DMRs are late-methylating.

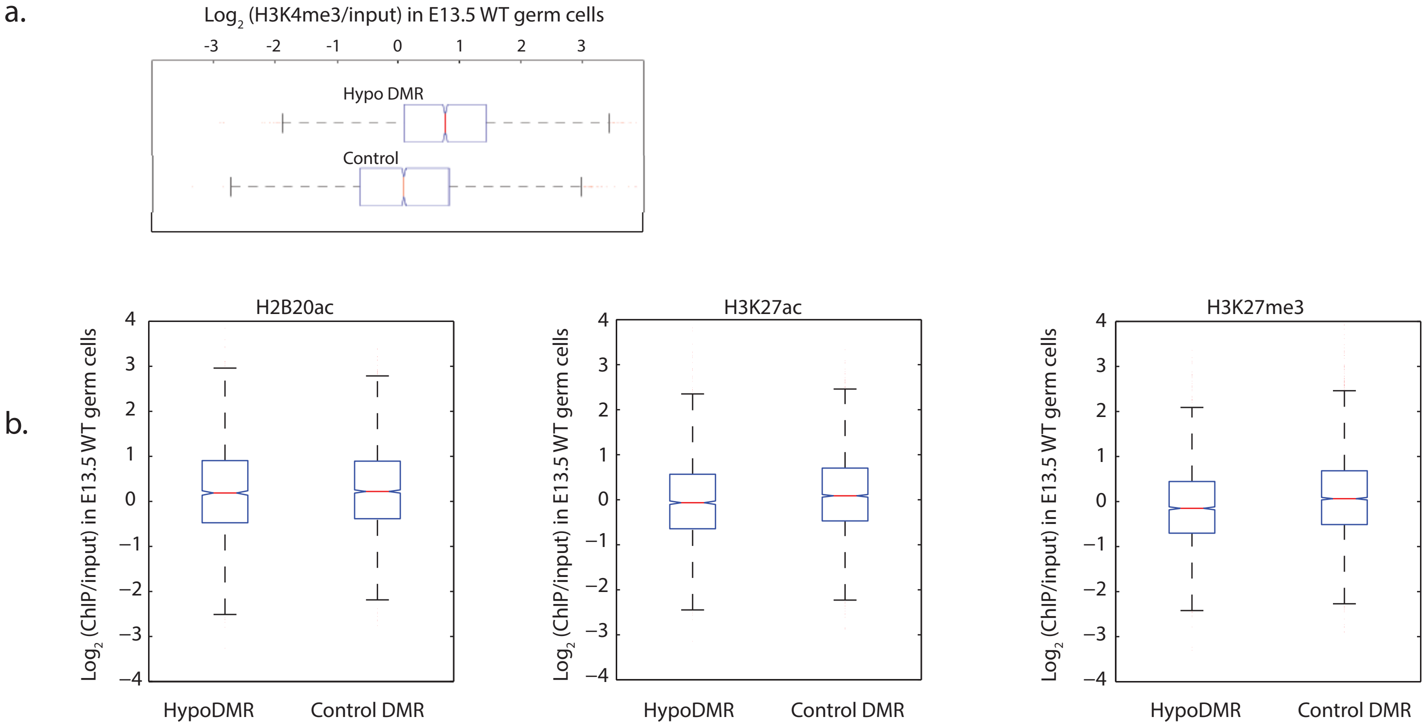

Supplementary Figure 11. Regions hypomethylated in *Morc1*$^{tg/tg}$ have elevated H3K4me3 at E13.5.
a-b, Abundance of ChIP-seq reads over control and hypomethylated DMRs are represented by boxplots. Each DMR corresponds to one point on the boxplot. (**a**) *Morc1*$^{tg/tg}$ DMRs have elevated H3K4me3 at E13.5, indicating that these regions are sites of transcriptional initiation at the onset of de novo methylation. (**b**) H2B20ac, K3K27ac and H3K27me3 abundances are similar over hypomethylated and control DMRs.

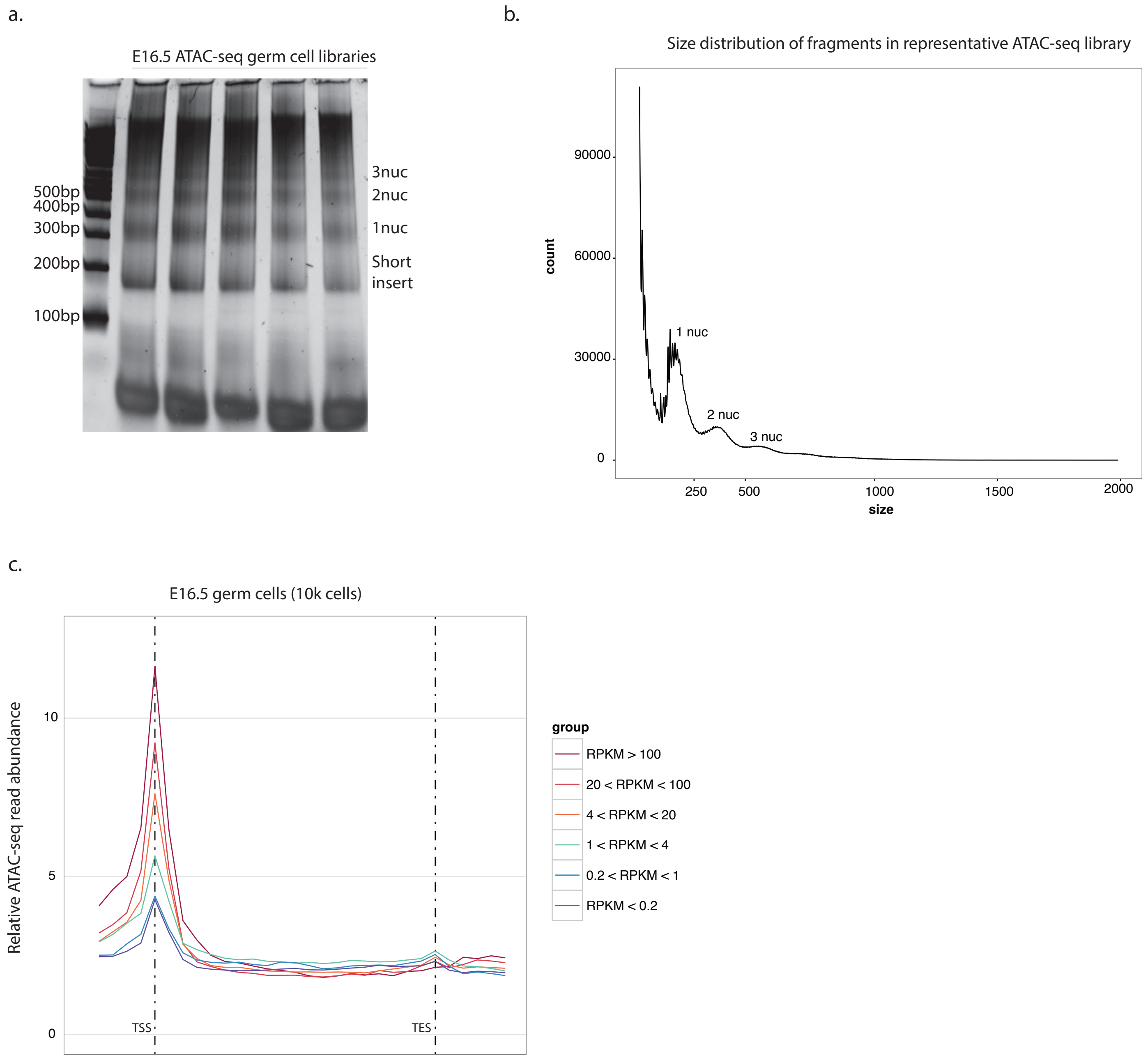

Supplementary Figure 12. Technical validation of the ATAC-seq method. a) ATAC-seq libraries using material from 10k embryonic germ cells produced an appropriate nucleosome-banding pattern, consistent with the tendency of primer insertion events to occur in nucleosome linker regions. **b**) The distribution of fragment sizes in the ATAC-seq library shows periodicity consistent with the 10.5bp pitch of DNA and the 180bp periodicity of nucleosome size. **c**) Metaplot of ATAC-seq reads relative to TSS in embryonic germ cells. A higher density of ATAC-seq reads is observed proximal to the TSS of the gene, with higher expressed genes showing greater ATAC-seq peaks. This confirms the potential of ATAC-seq as a method to find transcriptional start sites in embryonic germ cells.

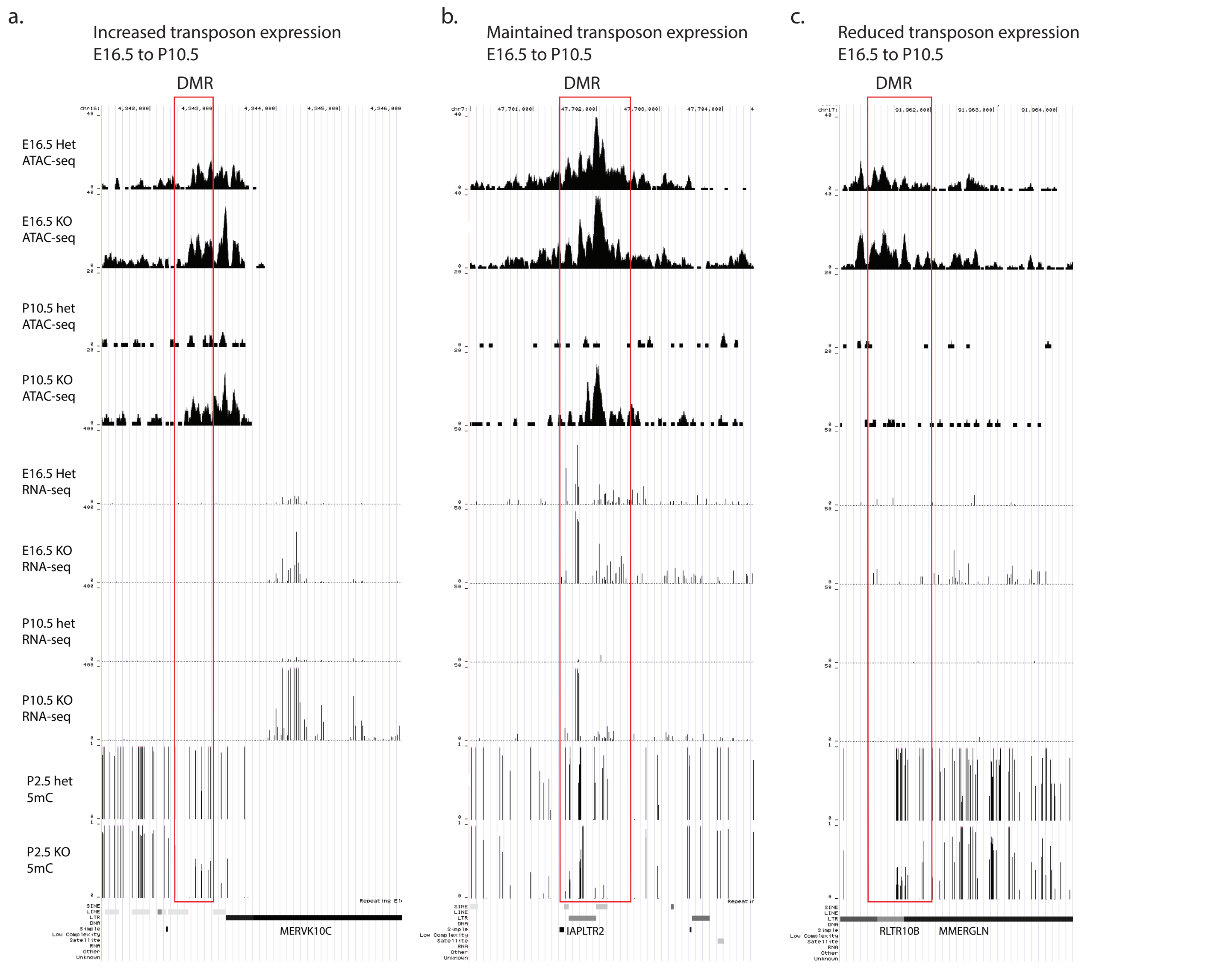

Supplementary Figure 13. ATAC-seq peaks follow the pattern of expression in the embryonic and postnatal germline. a-c, ATAC-seq , expression, and methylation data are shown at three loci. In all three loci, transposons are expressed at E16.5 and an ATAC-seq peak is present. Where transposon expression is sustained at P10.5 in *Morc1*$^{tg/tg}$ (**a**), (**b**), an peak of ATAC-seq reads is also present. Where transposon expression is silenced however, the ATAC-seq peak is lost (**c**).
